# Dual‐Stimuli Chromogenic Membranes for Optical Security: Photochromic and Halochromic Anti‐Counterfeiting Applications

**DOI:** 10.1002/smll.202507008

**Published:** 2025-08-04

**Authors:** Lin‐Ruei Lee, Yi‐Fan Chen, Po‐Xin Fan, Yu‐Chun Lin, Ming‐Hsuan Chang, Yu‐Chun Liu, Chun‐Chi Chang, Jiun‐Tai Chen

**Affiliations:** ^1^ Department of Applied Chemistry National Yang Ming Chiao Tung University Hsinchu 300093 Taiwan; ^2^ Center for Emergent Functional Matter Science National Yang Ming Chiao Tung University Hsinchu 300093 Taiwan

**Keywords:** anodic aluminum oxide (AAO), anti‐counterfeiting, photochromism, spiropyran, thiol‐yne chemistry

## Abstract

A dual‐stimuli chromogenic platform based on spiropyran‐functionalized anodic aluminum oxide (SP‐*t*‐AAO) membranes with reversible photochromic and halochromic switching is reported. Surface characterization by electronic images confirms the well‐preserved nanoporous morphology, while Energy‐dispersive X‐ray spectroscopy (EDS) scans reveal uniform grafting to micrometer depths. Grazing incidence X‐ray photoelectron spectroscopy (GIXPS) and time‐of‐flight secondary ion mass Spectrometry (TOF‐SIMS) further confirm successful surface chemical modification and pattern fidelity. Orthogonal functionalization is achieved via thiol‐yne chemistry and spatially controlled photopatterning using Chinese seasonal‐themed photomasks. Upon UV irradiation, the membrane exhibits a pale‐to‐magenta color change because of spiropyran‐merocyanine isomerization; acid exposure further switches the color from magenta to yellow. Both transitions are fully reversible under white light or base treatment. Video analysis reveals rapid halochromic switching kinetics (0.4–4 s), highlighting excellent optical responsiveness. The membrane also demonstrates the chelation toward heavy metal ions such as Cu^2+^ and Fe^2+^. Moreover, thermal cycling test shows moderate durability, and a 20‐day ambient test confirms excellent chromogenic stability. This work offers high‐speed responsiveness, spatial precision, and long‐term stability for anti‐counterfeiting and sensing applications.

## Graphical abstract

A dual‐stimuli chromogenic platform is developed based on spiropyran‐functionalized anodic aluminum oxide (SP‐t‐AAO) membranes, featuring reversible photochromic and halochromic switching. These membranes demonstrate rapid response, high spatial precision, and long‐term stability, making them highly suitable for anti‐counterfeiting and sensing applications.

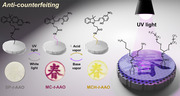

## 1 Introduction

Counterfeit and substandard products‐particularly in critical areas such as electronics, luxury merchandise, currency, pharmaceuticals, food, and confidential documents not only hinder global economic development but also pose serious threats to national security.^[^ ^1^, ^2^, ^3^, ^4^ ^]^ Based on available estimates, the global market for counterfeit products is valued at roughly 1 trillion USD dollars in 2022.^[^ ^4^, ^5^ ^]^ In response to the proliferation of counterfeiting goods, in recent years, there has been growing interest in the development of advanced materials and encryption strategies aimed at safeguarding data integrity and enhancing anti‐counterfeiting abilities.^[^ ^6^, ^7^, ^8^, ^9^ ^]^ Namely, a wide range of anti‐counterfeiting technologies have emerged, spanning from traditional encoding systems to more sophisticated, multi‐dimensional identifiers.^[^ ^6^ ^]^ These include 1D barcodes,^[^ ^10^ ^]^ 2D black‐and‐white QR codes,^[^ ^11^ ^]^ and 3D colorimetric codes,^[^ ^12^ ^]^ as well as 4D codes that incorporate dynamic, stimuli‐responsive features.^[^ ^13^ ^]^ Recent efforts also introduced laser‐written or volumetric color‐encoded materials to achieve 3D or 4D anti‐counterfeiting functionality in transparent or ceramic media.^[^ ^14^, ^15^ ^]^ Other approaches, such as laser‐induced holography,^[^ ^16^, ^17^, ^18^ ^]^ embedded watermarks,^[^ ^19^, ^20^ ^]^ and tunable luminescent patterns,^[^ ^20^ ^]^ have also found practical use in both industrial production and consumer applications.

Stimuli‐responsive materials, often referred to as smart or intelligent materials, are capable of altering their physical or chemical characteristics in response to external triggers.^[^ ^21^, ^22^ ^]^ Among stimuli‐responsive materials, chromogenic systems have gained particular attention as promising candidates for next‐generation anti‐counterfeiting applications because of their reversible and stimulus‐driven optical responses.^[^ ^4^, ^6^, ^23^ ^]^ These systems exhibit observable color changes in response to environmental triggers such as light,^[^ ^14^, ^15^, ^23^, ^24^, ^25^ ^]^ temperature,^[^ ^26^, ^27^ ^]^ pH values,^[^ ^28^, ^29^, ^30^ ^]^ water,^[^ ^31^ ^]^ mechanical force,^[^ ^32^ ^]^ and solvent polarity.^[^ ^33^, ^34^, ^35^ ^]^ Among them, spiropyran‐based molecular switches are particularly promising for anti‐counterfeiting applications because of their reversible transformation between a colorless spiropyran (SP) form and a colored merocyanine (MC) or protonated merocyanine (MCH) form.^[^ ^4^, ^36^, ^37^, ^38^, ^39^ ^]^ Upon UV light exposure, SP undergoes a ring‐opening isomerization to MC, which shows a vivid magenta or purple color. Further exposure to acidic vapors protonates MC into MCH, typically resulting in a yellow or orange hue.^[^ ^40^, ^41^ ^]^ These changes are reversible with white light or base vapor, enabling multicycle use.^[^ ^40^, ^41^ ^]^ Consequently, over the years, different research groups have devoted efforts to developing spiropyran‐based materials for anti‐counterfeiting applications.^[^ ^4^, ^42^ ^]^ For example, in previous studies, Duan et al. developed a dynamic fluorescent anti‐counterfeiting material by integrating spiropyran side groups into a poly[*p*‐(phenylene ethynylene)‐*alt*‐(thienylene‐ethynylene)] (PPTET) backbone. The system utilized both the photochromic behavior of spiropyran (undergoing ring‐opening upon UV exposure), and fluorescence resonance energy transfer (FRET) between the conjugated polymer and the merocyanine species.^[^ ^4^, ^43^ ^]^ In another case, Li and colleagues reported a fluorescent polymer capable of reversible photoswitching under visible light. In this design, a novel spiropyran‐based monomer (NSPMA) exhibiting negative photochromism was synthesized to enable fluorescence modulation through alternating visible light and thermal stimuli. Poly(methyl acrylate) (PMA) was selected as the polymer matrix to enhance the material's film‐forming properties, photostability, and overall processability.^[^ ^4^, ^44^ ^]^ Although previous studies have extensively demonstrated the use of spiropyran in dynamic anti‐counterfeiting systems, there has been limited progress in integrating spiropyran with highly commercialized robust inorganic substrates, such as anodic aluminum oxide (AAO),^[^ ^45^, ^46^ ^]^ to achieve durable, spatially programmable anti‐counterfeiting systems. Even in existing studies that employ AAO as a substrate for anti‐counterfeiting, the resulting pattern contrast tends to be relatively low,^[^ ^29^ ^]^ limiting visual clarity and authentication reliability. Therefore, in this study, we aim to bridge this gap by constructing a spatially patterned, dual‐responsive AAO‐based membrane system that integrates spiropyran as a reversible chromogenic element.

According to unique characteristics of AAO membranes, including high porosity, well‐ordered 3D nanoporous architecture, excellent thermal stability, and corrosion resistance, AAO membranes have been widely employed in diverse researches such as drug delivery, filtration, chemical sensing, and nanomaterial fabrication.^[^ ^47^, ^48^, ^49^, ^50^ ^]^ In addition, AAO materials have been widely utilized in industrial applications, such as components for mobile devices, automobiles, pipelines and aircrafts, as a result of their mechanical durability and corrosion resistance.^[^ ^51^, ^52^, ^53^, ^54^, ^55^, ^56^, ^57^ ^]^ Additionally, AAO surfaces exhibit excellent chemical modifiability, allowing for the stable covalent attachment of various functional groups through phosphonate and silane chemistries.^[^ ^58^ ^]^ Furthermore, AAO membranes are well‐suited for thiol‐yne click reactions, enabling the fabrication of anti‐counterfeiting patterns with high spatial resolution, rapid reaction kinetics, and high yields.^[^ ^59^, ^60^, ^61^, ^62^ ^]^ Accordingly, these features make AAO membranes highly promising for anti‐counterfeiting applications. If such functionalities are effectively integrated, AAO membranes may serve a broader role in commercial and industrial domains by meeting both security and performance demands.

Consequently, in this study, we present a spatially defined, dual‐stimuli chromogenic platform based on spiropyran‐functionalized AAO membranes (SP‐*t*‐AAO) that combines the advantages of spiropyran chemistry with the structural precision of AAO membranes. Our approach combines thiol‐yne click chemistry with photopatterning using seasonal‐themed Chinese character photomasks to achieve spatially selective surface functionalization on AAO membranes. Specifically, a photomask is used to guide the UV‐triggered thiol‐yne coupling reactions between terminal alkynes and either amine‐terminated (cysteamine hydrochloride) or fluorinated (*1H,1H,2H,2H*‐perfluorodecanethiol) thiols. Subsequent EDC/NHS coupling enables the selective grafting of spiropyran molecules onto the amine‐functionalized domains, yielding well‐defined chromogenic patterns with high spatial resolution on the AAO surface. Upon exposure to 365 nm UV light, the immobilized spiropyran undergoes ring‐opening to form the magenta‐colored merocyanine isomer on the AAO membrane (MC‐*t*‐AAO). Further exposure to acid vapors induces halochromic switching to the yellow protonated merocyanine state. These transitions are fully reversible with white light or TEA vapor, enabling robust photochromic and halochromic cycling. Using video‐based analysis with 0.01 s frame resolution, the halochromic response times are quantitatively determined under different acid vapors (TFA, HCl, CH_3_COOH, and HNO_3_), revealing fast switching behavior (0.4–4.3 s) and demonstrating the rapid responsiveness of the platform. The spatial confinement of color patterns is achieved by controlling the sequence of thiol‐yne modifications and photopatterning, enabling the fabrication of distinct Chinese seasonal character patterns (e.g., “Spring”, “Summer”, “Fall”, and “Winter”) for use as anti‐counterfeiting labels. Comprehensive surface characterization techniques, including grazing‐incidence X‐ray photoelectron spectroscopy (GIXPS), energy‐dispersive X‐ray spectroscopy (EDS), and time‐of‐flight secondary ion mass spectrometry (TOF‐SIMS), confirm chemical selectivity, functionalization in micrometer depth and spatial precision of the modifications. Moreover, optical analyses such as reflectance spectroscopy, CIE 1976 chromaticity mapping, pixel intensity standard deviation by histogram, and Canny edge detection reveal the dynamic and reversible chromogenic response of SP‐*t*‐AAO membranes. Environmental durability and mechanical robustness are also investigated. Specifically, the membranes retain their switching functions under humid vapor exposure and after surface abrasion by sandpaper, although their long‐term integrity is partially limited by the inherent brittleness of the AAO membrane. Thermal analysis using TGA also shows the onset of spiropyran decomposition above ≈100 °C, correlating with gradual contrast fading observed after thermal cycling. In addition, the metal ion sensing capability of the MC‐*t*‐AAO membrane is demonstrated via selective chelation with Cu^2+^ and Fe^2+^ ions, inducing visible color changes confined to the photoactivated regions. These selective interactions suggest potential applications in spatially encoded chemical sensing and data encryption. The integration of spiropyran molecules with nanoporous AAO membranes, combined with precise photopatterning through UV‐induced thiol‐yne chemistry, opens new strategies for developing multimodal and reversible anti‐counterfeiting materials, as well as broader applications in chemical sensing, optical data storage, and intelligent coatings.

## 2 Results and Discussion

**Figure 1:**
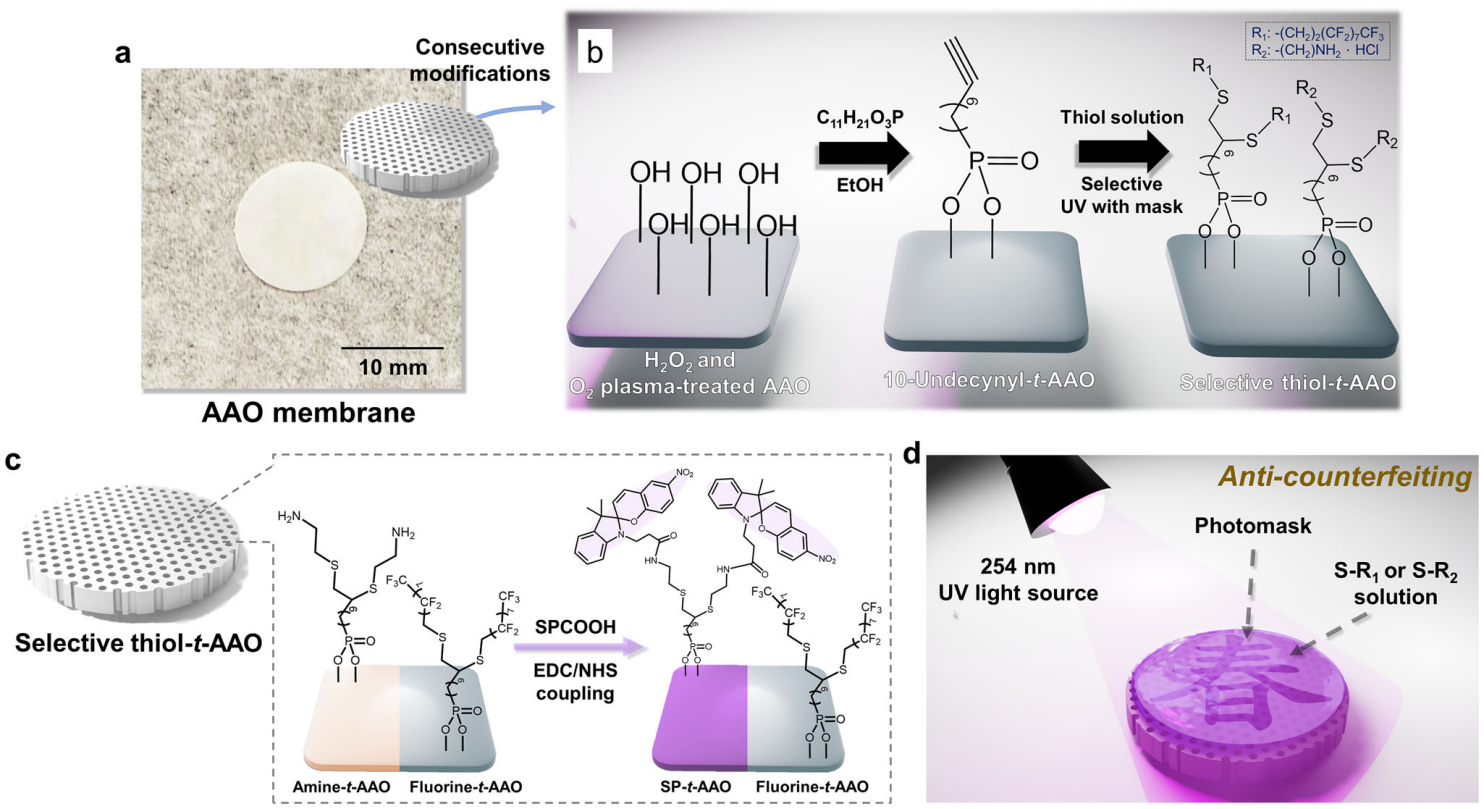
a) Real image of an anodic aluminum oxide (AAO) membrane. b) Schematic illustration of the preparation of a selectively thiol‐terminated AAO (thiol‐*t*‐AAO) membrane, featuring both amine‐ and fluorine‐terminated groups coexisting on the same membrane. c) Schematic representation of the fabrication of a spiropyran‐terminated AAO (SP‐*t*‐AAO) membrane from the selective thiol‐*t*‐AAO membrane through an EDC/NHS coupling reaction. d) Schematic illustration of the photopatterning process for producing a thiol‐*t*‐AAO membrane using a seasonal‐themed photomask, demonstrating potential for anti‐counterfeiting applications.

**Figure** **1**a presents the real image of the pristine AAO membrane, while Figure 1b schematically illustrates the sequential surface modification process. The surface is first treated with O_2_ plasma and H_2_O_2_ solution to generate a sufficient amount of hydroxyl (‐OH) groups. Subsequently, the membrane is reacted with a 10‐undecynylphosphonic acid solution in ethanol, leading to the grafting of terminal alkyne groups onto the AAO surface.^[^ ^63^ ^]^ As a result, the modified membrane, referred to as 10‐undecynyl‐*t*‐AAO, is obtained.^[^ ^61^ ^]^ In the subsequent step, a radical‐initiated thiol‐yne click reaction is employed to functionalize the AAO surface, in which thiol groups undergo radical‐mediated addition across the surface‐grafted terminal alkyne groups. Typically, this reaction involves UV irradiation in the presence of a photoinitiator to generate thiyl radicals from the thiol precursors. The generated thiyl radicals subsequently add across the carbon‐carbon triple bonds of the alkynyl‐functionalized AAO surface, yielding covalently bound terminal alkenyl sulfide moieties.^[^ ^59^, ^60^, ^61^ ^]^ This reaction proceeds efficiently under mild conditions and result in the robust covalent sulfur‐carbon bond formation. In this study, a photomask is used to selectively block UV light in specific regions, enabling spatially controlled thiol‐yne reactions between the thiol and alkyne groups on the AAO membrane, thereby producing a selectively patterned thiol‐*t*‐AAO surface. The spiropyran molecules (SP‐COOH) are subsequently grafted onto the AAO membrane via an EDC/NHS coupling reaction,^[^ ^64^, ^65^, ^66^ ^]^ as illustrated in Figure 1c. In this step, spiropyran is selectively grafted at sites where cysteamine hydrochloride (S‐R_2_) has been introduced.^[^ ^65^ ^]^ This selectivity arises because the carboxylic acid groups in the SP‐COOH molecules specifically react with the amine groups on the AAO surface, whereas the fluorinated groups from *1H,1H,2H,2H*‐perfluorodecanethiol (S‐R_1_) do not participate in this coupling reaction. In the EDC/NHS mechanism, EDC activates the carboxylic acid group of SP‐COOH to form an *O*‐acylisourea intermediate, which is then stabilized by NHS to generate an NHS ester.^[^ ^64^, ^66^ ^]^ This ester readily reacts with primary amines on the AAO surface to form a stable amide bond, completing the grafting process and producing the SP‐*t*‐AAO membrane. As illustrated in Figure 1d, To demonstrate the application of the SP‐*t*‐AAO membrane, a seasonal‐themed photomask (“Spring”, “Summer”, “Fall”, and “Winter”) is placed on the membrane, which is then exposed to 254 nm UV light to spatially control the modification sites of S‐R_1_ and S‐R_2_.^[^ ^59^, ^61^ ^]^ By altering the sequence of surface modifications with S‐R_1_ and S‐R_2_, different spatial patterns can be achieved.^[^ ^59^ ^]^ This variation in modification order results in two distinct and opposite patterns upon subsequent grafting of SP‐COOH.^[^ ^59^ ^]^

In this study, the photo‐responsive and photochromic compound SP‐COOH is synthesized via a two‐step process,^[^ ^65^, ^67^ ^]^ as illustrated in Figure S1a (Supporting Information). Specifically, 2,3,3‐trimethylindolenine is first reacted with 3‐iodopropionic acid to form a carboxylic acid‐functionalized indolenine intermediate, which is then condensed with 5‐nitrosalicylaldehyde to introduce the pyran moiety. Figure S1b (Supporting Information) presents the ^1^H NMR spectrum of SP‐COOH, which shows characteristic chemical shifts consistent with the proposed structure, including signals corresponding to the indolenine and pyran moieties. Figure S2 (Supporting Information) displays the ESI‐MS spectra acquired in both positive and negative ionization modes, revealing a molecular ion peak that matches the calculated molecular weight of SP‐COOH (mass error < 1 ppm).^[^ ^67^ ^]^ Therefore, the ^1^H NMR and mass spectrometric data provide the evidence for the successful synthesis and structural integrity of the target compound. Furthermore, UV–vis spectroscopy is employed to investigate the photochromic behavior of SP‐COOH.^[^ ^65^ ^]^ As shown in the UV–vis spectrum (Figure S3a, Supporting Information), SP‐COOH exhibits a characteristic absorption peak at ≈335 nm. Upon UV irradiation, SP‐COOH undergoes a ring‐opening isomerization to form MC‐COOH, resulting in the appearance of a new absorption peak at ≈596 nm. The residual absorption at ≈335 nm after UV exposure is attributed to incomplete conversion of SP‐COOH to MC‐COOH.^[^ ^65^ ^]^ Additionally, as the irradiation time increases, the absorbance at ≈596 nm gradually rises (Figure S3b, Supporting Information), indicating the progressive formation of MC‐COOH in solution. After 180 s of UV exposure, the absorbance appears to reach a plateau, suggesting that the photoisomerization has approached saturation under the given conditions. More specifically, as shown in Figure S3c (Supporting Information), the natural logarithm of the absorbance at ≈596 nm is plotted to determine the rate constant for the photoinduced transformation of SP‐COOH to MC‐COOH. Assuming a first‐order reaction, the rate constant for the ring‐opening process is estimated to be 0.0167 s^−1^.^[^ ^68^ ^]^

To further confirm that the surface functionalization process does not affect the structural integrity of the AAO templates, scanning electron microscopy (SEM) imaging on both pristine and modified AAO membranes are conducted.^[^ ^61^ ^]^ As shown in Figure S4a–h (Supporting Information), top‐view (0°) and cross‐sectional (90°) SEM images of pristine AAO, amine‐*t*‐AAO, fluorine‐*t*‐AAO, and SP‐*t*‐AAO reveal that the well‐aligned nanopore arrays and vertical pore structures are well preserved after consecutive functionalization. Statistical analyses of the pore diameter distributions (Figure S4i, Supporting Information) suggest that the median pore size remains nearly unchanged (161.5–162.8 nm) across all samples, indicating that the small‐molecule grafting does not cause significant pore deformation, blockage, or collapse.^[^ ^61^ ^]^ These results confirm that the chemical modifications proceed without compromising the original nanostructures of the AAO membranes. Besides, to understand the surface properties of AAO membranes after consecutive modifications, static water contact angle (WCA) measurements and grazing incidence X‐ray photoelectron spectroscopy (GIXPS) with 15° X‐ray incidence are conducted.^[^ ^61^, ^65^, ^69^ ^]^ As shown in Figure S5 (Supporting Information), the water contact angle significantly changes depending on the surface functional groups introduced on the AAO membrane. The alkyne‐modified AAO membrane (alkyne‐*t*‐AAO) exhibits a moderately hydrophobic surface with a WCA of ≈120°, which decreases to ≈90° upon amine functionalization (amine‐*t*‐AAO) because of the hydrophilic nature of ‐NH_2_ groups. Besides, immobilizing of fluorinated groups significantly enhances surface hydrophobicity, reaching ≈150° (fluorine‐*t*‐AAO).^[^ ^61^ ^]^ This increase in hydrophobicity is attributed to the low surface energy and high water repellency provided by the densely packed perfluorinated alkyl chains on the surface.^[^ ^61^ ^]^ After grafting with spiropyran molecules (SP‐*t*‐AAO) from alkyne‐saturated surface, the surface maintains hydrophobicity with a WCA ≈107°. Upon UV‐triggered ring‐opening to the merocyanine form (MC‐*t*‐AAO), the surface exhibits significantly increased hydrophilicity, with the water contact angle sharply decreasing to ≈15°. This pronounced change demonstrates distinct photo‐responsive wetting behavior, attributed to the emergence of zwitterionic merocyanine species on the surface.^[^ ^70^ ^]^

**Figure 2:**
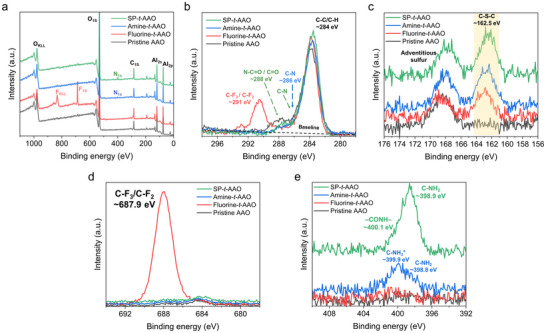
Grazing incidence X‐ray photoelectron spectroscopy (GIXPS) spectra of AAO membranes after sequential chemical surface modifications: a) survey scans; high‐resolution scans of b) C 1s, c) S 2p, d) F 1s, and e) N 1s regions for alkyne‐*t*‐AAO, fluorine‐*t*‐AAO, amine‐*t*‐AAO, and SP‐*t*‐AAO membranes.

The XPS spectra on modified AAO membranes further confirm the successful chemical modifications, as shown in **Figure** **2**. The GIXPS spectra of survey scan (Figure 2a) show distinct elemental signals corresponding to the introduced functional groups. Overall, the peaks observed at binding energies ≈74.0, 118.0, and 530.0 eV correspond to the Al 2p, Al 2s, and O 1s core levels of the AAO membranes, respectively. It is noteworthy that the peaks at ≈400.0 eV are attributed to nitrogen (N 1s) signals after amine and spiropyran modification, and the peak at ≈688.0 eV is represented by fluorine (F 1s) signals after the modification of fluorinated thiol.^[^ ^61^, ^65^ ^]^ Furthermore, the high‐resolution scanning of the sample at an energy resolution of 0.1 eV in the C 1s, S 2p, F 1s, and N 1s regions is also used to characterize the samples after modifications (Figure 2b–e). High‐resolution C 1s spectra (Figure 2b) substantiate the sequential surface modifications. In the fluorine‐*t*‐AAO membrane, a peak at ≈291.0 eV is characteristic of C‐F bonds from the grafted perfluoroalkyl chains.^[^ ^71^ ^]^ After spiropyran attachment (SP‐*t*‐AAO), a new component appears at ≈288.0 eV, assigned to carbonyl/ether carbons (N─C═O and C─O) within the spiropyran structure, along with a signal at ≈286.0 eV attributed to C‐N bonds.^[^ ^68^, ^72^ ^]^ The same 286.0 eV feature is also present on the amine‐*t*‐AAO membrane, reflecting the introduction of amino groups through thiol‐yne reaction. Collectively, these binding energy signatures confirm the successful grafting of fluoro‐thiol, spiropyran, and amino‐thiol functionalities onto the AAO surfaces. Furthermore, the S 2p spectra (Figure 2c) clearly indicate the presence of C─S─C groups (≈162.5 eV), arising from successful thiol‐yne click reactions.^[^ ^73^ ^]^ The F 1s high‐resolution spectrum (Figure 2d) reveals a strong fluorine signal (≈687.9 eV) unique to fluorine‐*t*‐AAO, validating fluorinated surface modifications. High‐resolution N 1s spectra (Figure 2e) resolve three distinct nitrogen environments. A peak at ≈400.1 eV is assigned to amide nitrogen (─CONH─) and appears exclusively after spiropyran is covalently attached to the amine‐*t*‐AAO surface,^[^ ^74^ ^]^ confirming successful EDC/NHS coupling. The band at ≈399.9 eV corresponds to protonated amines (C─NH_3_⁺) present on the amine‐*t*‐AAO membrane, whereas the signal at ≈398.9 eV arises from neutral amines (C─NH_2_) represented by spiropyran grafting to form the SP‐*t*‐AAO membrane.^[^ ^68^ ^]^ The emergence of the amide peak which is concurrent with the attenuation of the protonated‐amine band demonstrates efficient conversion of surface amines into amide linkages during spiropyran immobilization. Furthermore, to further evaluate the spatial distribution and depth of the grafted molecules, the energy‐dispersive X‐ray spectroscopy (EDS) analysis is carried out on the modified membranes.^[^ ^75^ ^]^ As shown in Figure S6 (Supporting Information), the EDS spectrum of fluorine‐*t*‐AAO confirms the presence of fluorine (F), along with signals from aluminum (Al), oxygen (O), and carbon (C). Compared to above‐mentioned surface‐sensitive techniques, XPS, EDS enables compositional profiling over a few micrometers in depth.^[^ ^75^, ^76^ ^]^ The detection of fluorine throughout the probed volume supports the conclusion that the fluorinated molecules are uniformly coated within the nanopores of the AAO membrane through thiol‐yne click chemistry, rather than confined to only the external surface.^[^ ^61^ ^]^ Overall, these analytical results confirm precise chemical control and successful functionalization of AAO membranes.

In addition to confirming chemical composition of the patterned AAO membrane using GIXPS and EDS, TOF‐SIMS analyses are further employed to verify the spatially resolved surface functionalization of the SP‐*t*‐AAO and fluorine‐*t*‐AAO regions of the patterned AAO.^[^ ^77^ ^]^ Figure S7 (Supporting Information) shows the TOF‐SIMS spectra collected under both positive and negative ion modes for these two regions in the same membrane. In the positive ion mode (Figure S7a, Supporting Information), the SP‐*t*‐AAO region exhibits a characteristic signal at m/z 27 corresponding to C_2_H_3_^+^, which is attributed to the fragmentation of the aromatic ring structure of the spiropyran moiety. In contrast, the fluorine‐*t*‐AAO region displays a prominent peak at m/z 69 assigned to CF_3_^+^, a typical fragment from the perfluorinated thiol.^[^ ^77^ ^]^ In the negative ion mode (Figure S7b, Supporting Information), the SP‐*t*‐AAO region shows signals at m/z 16 and 26, corresponding to O^−^ and CN^−^, respectively, both derived from the spiropyran molecular structure. Additionally, peaks at m/z 63 (PO_2_^−^) and 79 (PO_3_^−^) are observed in both SP‐*t*‐AAO and fluorine‐*t*‐AAO spectra, confirming the presence of phosphonic acid anchoring groups resulting from the reaction with 10‐undecynylphosphonic acid on the AAO surface.^[^ ^78^ ^]^ For the fluorine‐*t*‐AAO region, F^−^ (m/z 19) and CF_3_^−^ (m/z 69) signals dominate the negative ion mode, further verifying the successful grafting of the fluorinated thiol.^[^ ^77^, ^78^ ^]^ The presence of both phosphonate‐derived ions and thiol‐specific fragments in the respective regions confirms that the thiol‐yne click reactions proceed in a spatially selective manner. The results from TOF‐SIMS analyses, in conjunction with EDS and GIXPS data, provide evidence that the surface functionalization proceeds in a spatially selective manner on the surface, while also achieving uniform functionalization in depth within the nanopores, enabling the successful fabrication of the patterned AAO membranes.

**Figure 3:**
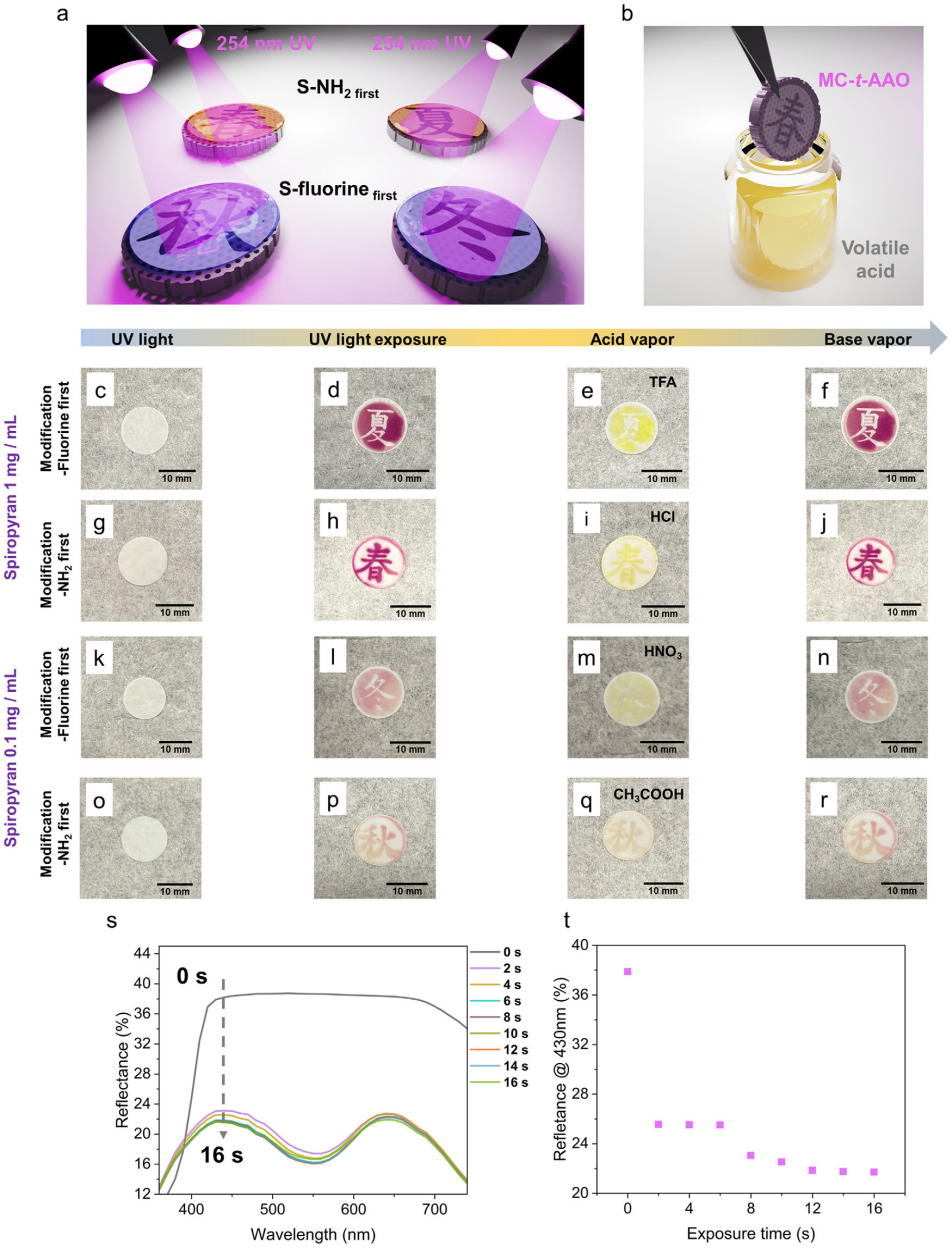
a) Schematic illustration of selective thiol‐yne surface modifications with seasonal‐themed photopatterning under 254 nm UV irradiation. b) Schematic illustration of the color change of a MC‐*t*‐AAO membrane upon exposure to various volatile acids. c–f) Real images of the c) “Summer” pattern before UV irradiation, d) after UV irradiation, e) after TFA vapor exposure, and f) after TEA vapor exposure (f). g–j) Real images of the g) “Spring” pattern before UV irradiation, h) after UV irradiation, i) after HCl vapor exposure, and j) after TEA vapor exposure. k–n) Real images of the k) “Winter” pattern before UV irradiation, l) after UV irradiation, m) after HNO_3_ vapor exposure, and n) after TEA vapor exposure. o–r) Real images of the o) “Fall” pattern before UV irradiation, p) after UV irradiation, q) after CH_3_COOH vapor exposure, and r) after TEA vapor exposure. s) Reflectance spectra showing the optical changes of an SP‐*t*‐AAO membrane upon exposure to 365 nm UV light over time (0–16 s). t) Reflectance intensity at ≈430 nm plotted as a function of UV exposure time (0–16 s), quantifying the photo‐responsive behavior of the SP‐*t*‐AAO membrane.

To explore the practical applicability of the AAO membrane that is modified with spiropyran, spatially controlled photopatterning is demonstrated using seasonal‐themed photomasks under 254 nm UV irradiation, highlighting the potential for anti‐counterfeiting applications. As illustrated in **Figure** **3**a, the AAO membranes are sequentially modified through thiol‐yne click reactions, where either amino‐terminated (S─NH_2_) or fluorinated (S–fluorine) thiols could be introduced as the first modification step.^[^ ^59^, ^60^, ^61^, ^62^, ^77^ ^]^ This chemoselective functionalization enables the following selective grafting of spiropyran molecules (SP‐COOH) through EDC/NHS coupling to amino thiol‐modified regions, thus allowing for patterned functionalization.^[^ ^79^ ^]^ Therefore, the sequence‐defined surface modification leads to the formation of the SP‐*t*‐AAO membrane with well‐defined spatial functionalization. When the SP‐*t*‐AAO membrane is irradiated with 365 nm UV light, the spiropyran units undergo ring‐opening isomerization to form the colored merocyanine isomer exclusively in the amino thiol‐modified regions defined by the sequential thiol‐yne reactions, resulting in distinct magenta‐colored patterns representing the four seasons (“Spring”, “Summer”, “Fall”, and “Winter”) and generating the MC‐*t*‐AAO membrane. This photoisomerization involves cleavage of the spiro C─O bond, allowing for π‐electron delocalization across the conjugated system.^[^ ^41^, ^80^ ^]^ Besides, Figure 3b depicts a conceptual demonstration of the MC‐*t*‐AAO membrane's function, where a patterned MC‐*t*‐AAO membrane is exposed to volatile acidic vapor, triggering a visible color change.^[^ ^81^ ^]^ Figure 3c–r illustrates the sequential optical responses of SP‐*t*‐AAO membranes subjected to UV irradiation and the following acid/base vapor treatment. It should be mentioned again that the coloration from the SP‐*t*‐AAO to the MC‐*t*‐AAO membrane originates from the selective surface grafting of spiropyran (SP‐COOH) onto amino‐functionalized regions. As a result, the specific sequence of thiol‐yne surface modifications, whether introducing NH_2_ or fluorinated groups first, can generate two distinct and opposite color patterns, as demonstrated in Figure 3d,h,l,p. The intensity of the coloration, however, varies depending on the density of spiropyran grafting.^[^ ^42^ ^]^ In particular, the patterns in Figure 3l,p appear lighter than those in Figure 3d,h, which can be attributed to the lower spiropyran concentration used during modification (0.1 versus 1 mg/mL, respectively). The reduced concentration leads to less spiropyran incorporation during the EDC/NHS coupling reaction, resulting in diminished color intensity.^[^ ^82^, ^83^ ^]^ The photoactivated MC‐*t*‐AAO membranes are then exposed to vapors of various volatile acids to evaluate their halochromic response. In Figure 3e,i,m,q, the purple patterns undergo a clear shift toward yellow upon exposure to TFA, HCl, HNO_3_, and CH_3_COOH, respectively, suggesting protonation of the merocyanine to yield the protonated merocyanine on AAO membranes (MCH‐*t*‐AAO).^[^ ^65^ ^]^ Notably, in the case of exposure to CH_3_COOH (Figure 3q), the resulting coloration appears as a mixture of purple and yellow. This intermediate tone is because of the relatively weak acidity of acetic acid, which limits the extent of merocyanine protonation, resulting in only partial conversion to protonated merocyanine.^[^ ^65^ ^]^ Reversibility is further confirmed by exposing the acid‐treated AAO membranes to TEA vapor. In all cases (Figure 3f,j,n,r), the original magenta patterns are restored, demonstrating the complete and reversible interconversion between merocyanine and protonated merocyanine states under vapor‐phase acid/base cycling. This robust responsiveness confirms the reversible interconversion among spiropyran, merocyanine, and protonated merocyanine states on the AAO membrane.

To quantify the color change after the membranes are exposed to UV light and acid vapors, contrast analyses are used.^[^ ^84^ ^]^ Figure S8 (Supporting Information) shows pixel intensity histograms obtained from optical images of the SP‐*t*‐AAO membranes after initial photoactivation by 365 nm UV irradiation and the subsequent acid vapor exposure. The distributions represent the weighted color intensity of each pixel across the membrane area and provide insight into the uniformity and magnitude of the visual response. In all four cases (TFA, HCl, HNO_3_, and CH_3_COOH), UV light exposure (orange curves) yields a broad distribution of pixel intensities with high standard deviation (σ), reflecting the presence of distinct but spatially varied magenta merocyanine domains. In Figure S8a (Supporting Information), for example, the UV‐irradiated TFA sample exhibits a broad intensity distribution with σ = 50.1, indicating a strong heterogeneous color contrast across the patterned region. Upon exposure to acid vapors (green curves), a shift in pixel intensity is observed toward the higher‐intensity region (180–250 range), accompanied by a sharp reduction in the standard deviation (σ), which suggests a more homogeneous and lighter color appearance due to merocyanine‐to‐protonated merocyanine conversion. For TFA and HCl (Figure S8a,b, Supporting Information), the standard deviation decreases drastically to σ = 7.6 and σ = 8.1, respectively, indicating a complete and uniform protonation of the merocyanine form into the protonated merocyanine state. In contrast, the relatively small standard deviation changes observed in HNO_3_ and CH_3_COOH cases (Figure S8c,d, Supporting Information), with σ decreasing from 13.7 to 4.6 and from 13.4 to 7.4, respectively, are primarily attributed to the lower concentration (0.1 mg mL^−1^) of spiropyran used during the surface modification step. The limited grafting density of spiropyran results in a reduced colorimetric response and less pronounced optical contrast between the UV‐exposed and acid‐treated states.^[^ ^82^ ^]^ Notably, the CH_3_COOH‐treated sample exhibits a slightly higher standard deviation than the HNO_3_ case after acid exposure, which is attributed to the incomplete conversion of the merocyanine form to the protonated merocyanine form.^[^ ^65^ ^]^ Because of the relatively weak acidity of acetic acid (pK_a_ ≈4.76),^[^ ^85^ ^]^ a portion of the original purple merocyanine color remains visible, producing a mixed or intermediate color state and contributing to broader pixel intensity distribution. These findings suggest that the contrast of anti‐counterfeiting patterns generated on SP‐*t*‐AAO membranes can be effectively tuned by adjusting the spiropyran concentration during the surface grafting step.

Time‐dependent reflectance measurements are also conducted to characterize the kinetics of the spiropyran‐to‐merocyanine photoisomerization under 365 nm UV irradiation.^[^ ^65^, ^86^ ^]^ As shown in Figure 3s, a distinct decrease in reflectance at ≈430 and ≈640 nm is observed with increasing UV exposure time, indicating the formation of the merocyanine form on the membrane surface. In the initial state, the SP‐*t*‐AAO membrane exhibits high reflectance across the visible range because of the non‐absorbing nature of the closed‐ring spiropyran form. Upon UV irradiation, the increasing MC population leads to the apparent reflectance at ≈430 and ≈640 nm.^[^ ^65^ ^]^ The conversion kinetics, plotted in Figure 3t, indicate rapid photoisomerization within the first few seconds, reaching a photostationary state by 12–16 s. To quantify the chromogenic response more precisely, frame‐by‐frame image extraction at 0.01 s intervals is performed to track the color transition from the purple merocyanine form to the yellow protonated merocyanine form. The sequential images are presented in Figure S9a–h (Supporting Information), demonstrating the real‐time halochromic behavior upon exposure to TFA, HCl, CH_3_COOH, and HNO_3_ vapors. The response times are quantitatively determined by identifying the frame at which the complete color change occurs, and the comparative results are summarized in Figure S9i (Supporting Information). The data reveal that TFA induces the fastest transition, followed by HCl, CH_3_COOH, and HNO_3_, which is consistent with differences in vapor pressure and acidity among the acids. In particular, the commercial‐grade HNO_3_ (68%–70% in water) exhibits slower protonation kinetics because of its relatively low volatility and weak fuming behavior. The results demonstrate that the halochromic response of MC‐*t*‐AAO membranes is tunable based on the properties of the surrounding acid vapor.

**Figure 4:**
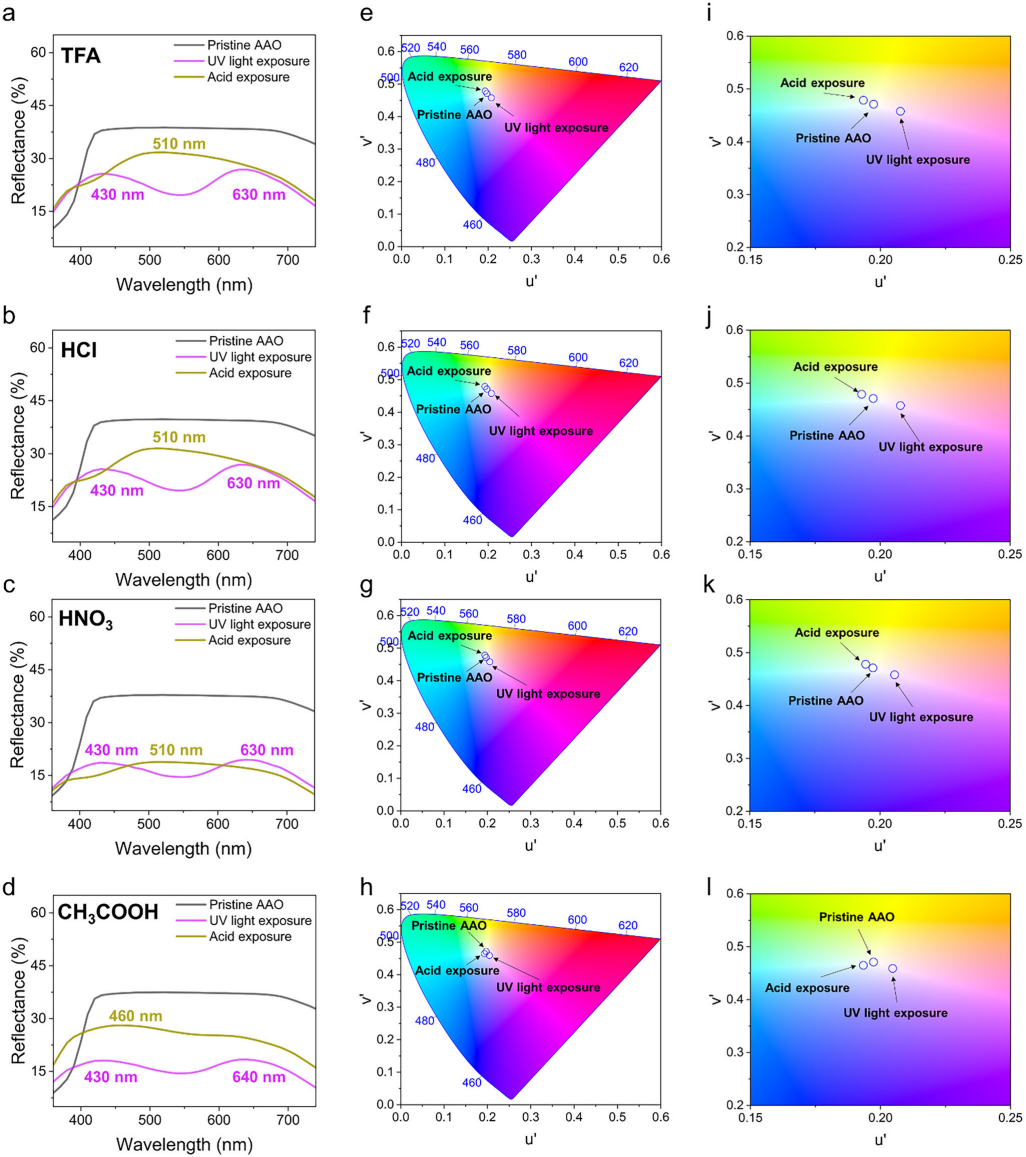
a–d) UV–vis reflectance spectra of SP‐*t*‐AAO membranes exposed sequentially to 365 nm UV light and acid vapors (TFA, HCl, HNO_3_, and CH_3_COOH). e–h) CIE 1976 chromaticity diagrams derived from the reflectance spectra (TFA, HCl, HNO_3_, and CH_3_COOH). i–l) Enlarged chromaticity diagrams derived from the reflectance spectra (TFA, HCl, HNO_3_, and CH_3_COOH).

To further investigate the light‐ and acid‐induced optical transitions of SP‐*t*‐AAO membranes, reflectance spectra and CIE 1976 (u’, v’) chromaticity analyses are performed, as shown in **Figure** **4**. The CIE 1976 chromaticity diagram, an improvement over the original CIE 1931 model, provides a more uniform chromaticity scale, making it suitable for quantitatively analyzing subtle color changes in photochromic and halochromic materials.^[^ ^29^, ^87^, ^88^ ^]^ The detailed chromaticity coordinate calculation process, including equations and spectral parameters, is described in Note S1 (Supporting Information).^[^ ^89^ ^]^ Figure 4a shows the reflectance spectra of the case treated with TFA, illustrating the three optical states: the SP‐*t*‐AAO surface (gray), MC‐*t*‐AAO surface (magenta), and the protonated MCH‐*t*‐AAO surface state after acid vapor treatment (yellow). Upon UV irradiation, a significant peak in reflectance ≈430 and 630 nm appears, corresponding to the formation of merocyanine chromophore, which shows the magenta color. After the exposure to TFA, a new peak near 510 nm becomes dominant, confirming the conversion from merocyanine to protonated merocyanine through protonation.^[^ ^65^ ^]^ Figure 4e presents the corresponding CIE 1976 chromaticity diagram derived from the reflectance data in Figure 4a. The color coordinate of the pristine membrane lies near the center, then shifts toward the magenta region after UV exposure, and finally moves into the yellow region after acid exposure. The color trajectory is long and well‐defined, indicating a strong and complete transition from the spiropyran form to the merocyanine form, followed by protonation to the protonated merocyanine state. Figure 4i shows an enlarged view of the chromaticity shift in (u’, v’) space, where the directional arrow highlights the significant movement from the spiropyran to merocyanine and then to protonated merocyanine state. The sharp displacement reflects TFA's high protonating efficiency and strong vapor pressure. Figures 4b,f,j depict the same sequence for HCl‐treated membranes. As shown in Figure 4b, UV light triggers similar merocyanine formation with a clear 430 nm band and 630 nm shoulder. Upon HCl exposure, a strong 510 nm peak again indicates protonated‐merocyanine formation. Figure 4f shows the CIE 1976 coordinate shift from pristine to magenta to yellow, similar to TFA. The enlarged chromaticity map in Figure 4j confirms a substantial color shift, with a trajectory comparable to that observed with TFA, indicating similar vapor‐driven response behavior. Figure 4c,g,k presents the data for HNO_3_ exposure. Compared to Figure 4a,b, Figure 4c shows the formation of relatively minor reflectance features at ≈430 and ≈630 nm after UV irradiation, followed by a relatively weak reflectance maximum at ≈510 nm after acid treatment. This result suggests that a lower concentration of surface‐bound spiropyran participated in the photoisomerization and further protonation process.^[^ ^82^ ^]^ In Figure 4g, the chromaticity trajectory is less pronounced, and Figure 4k displays a shorter vector displacement in the (u’, v’) space. These results are consistent with a lower initial concentration of grafted spiropyran on the AAO membrane,^[^ ^82^ ^]^ leading to limited merocyanine formation and consequently reduced conversion to protonated merocyanine upon HNO_3_ exposure, especially when compared to the more pronounced responses observed with TFA and HCl. In the last case, Figure 4d,h,l corresponds to CH_3_COOH treatment. It is worthy to note that Figure 4d reveals weaker spectral changes with incomplete suppression of the merocyanine bands at ≈430 and ≈630 nm and less pronounced development of the protonated merocyanine peak at ≈510 nm after MC‐*t*‐AAO membrane is exposed to CH_3_COOH. Figure 4h shows that the chromaticity shift remains between the magenta and yellow regions, and Figure 4l confirms that the shortest vector trajectory is observed in this case. This incomplete transition is attributed to the combined effects of a lower surface concentration of grafted spiropyran and the weak acidity of CH_3_COOH, leading to partial merocyanine‐to‐protonated merocyanine conversion.^[^ ^65^, ^82^ ^]^

**Figure 5:**
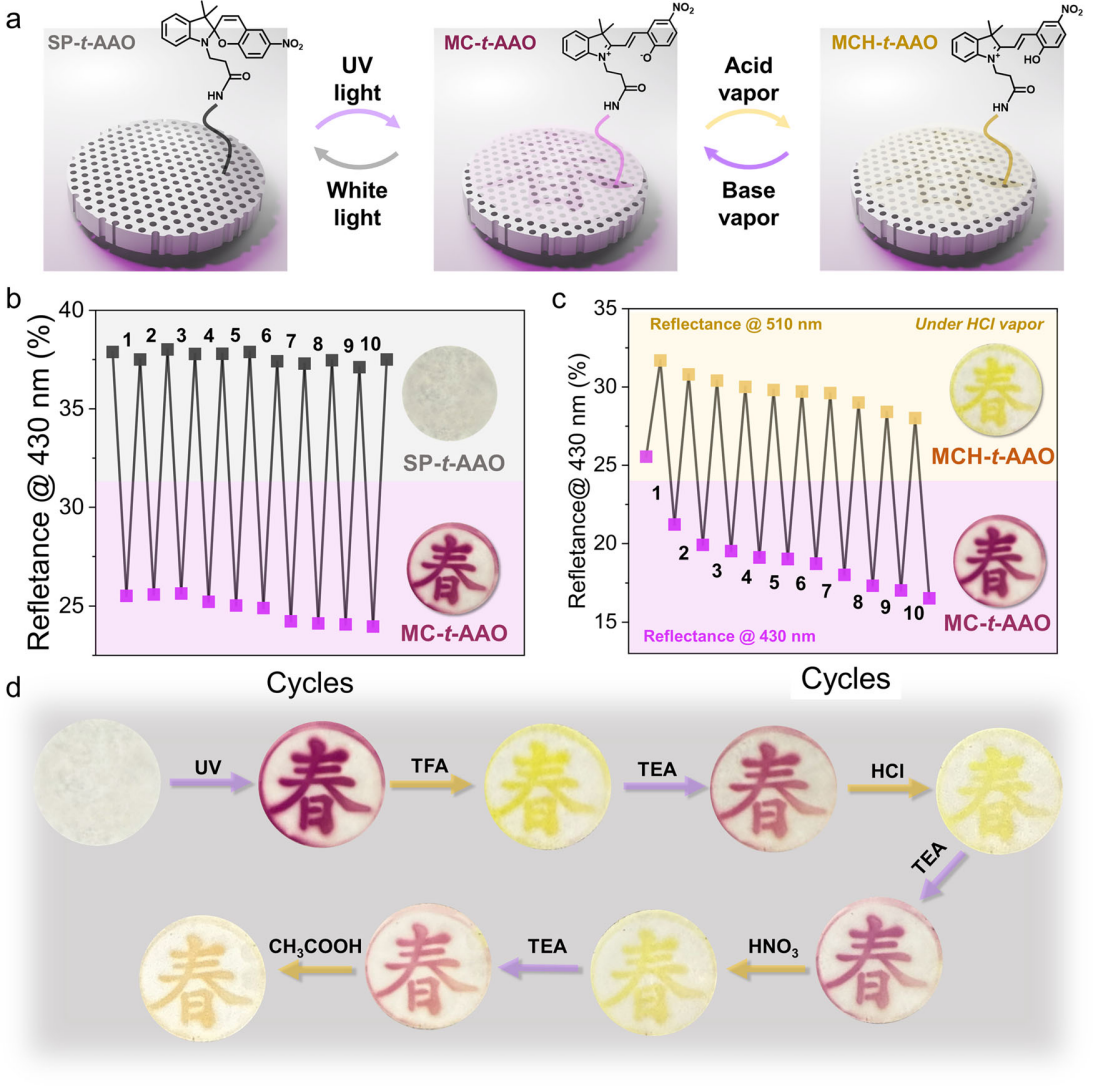
a) Schematic illustration of the molecular switching mechanism on the AAO surface, showing transitions among the spiropyran, merocyanine, and protonated merocyanine states. b) Reversibility test of SP‐*t*‐AAO under repeated UV on/off cycles. c) Reversibility test under alternating HCl and TEA vapor treatments. Reflectance at 430 and 510 nm is monitored over 10 cycles. d) Reusability test of a single SP‐*t*‐AAO membrane under repeated acidic and TEA vapor exposures using different acids (TFA, HCl, HNO_3_, and CH_3_COOH).

To evaluate the dynamic response and cycling stability of the SP‐*t*‐AAO membrane system, the membrane's reversibility is systematically investigated under repeated UV irradiation and acid/base exposure, as well as its reusability under sequential acid vapors using a single sample,^[^ ^65^ ^]^ as shown in **Figure** **5**. Figure 5a illustrates the reversible chemical transformations between the spiropyran, merocyanine, and protonated merocyanine forms immobilized on the surface of AAO membranes. Upon 365 nm UV irradiation, the colorless spiropyran form undergoes ring‐opening isomerization to generate the highly conjugated and zwitterionic merocyanine form, which imparts a vivid magenta color. Subsequent exposure to acidic vapor (e.g., HCl) protonates the merocyanine form, yielding the protonated merocyanine species with reduced conjugation and a corresponding yellow appearance.^[^ ^41^ ^]^ This reversible transformation forms the basic mechanism for the membrane's optical switching behavior. For examining reversibility between SP‐*t*‐AAO and MC‐*t*‐AAO membranes, Figure 5b presents a reversibility test of the SP‐*t*‐AAO membrane over six UV light on/off cycles. The membrane is initially colorless (spiropyran state), and upon UV irradiation, it turns magenta (merocyanine state).^[^ ^23^ ^]^ This reversible spiropyran‐to‐merocyanine photochromic transformation is further evaluated through multiple UV on/off cycles, as shown in Figure 5b. Over 10 switching cycles, the patterned magenta coloration associated with the merocyanine form could be consistently regenerated, demonstrating good photochromic reversibility. A slight decrease in reflectance intensity, however, is observed over repeated cycles (from 25.5 to 23.9%), suggesting the onset of photo fatigue or partial structural degradation of the surface‐bound spiropyran.^[^ ^65^, ^90^, ^91^ ^]^ This minor decrease may also be attributed to photobleaching that reduce the efficiency of spiropyran‐to‐merocyanine interconversion over time.^[^ ^23^, ^65^, ^90^, ^91^ ^]^ Figure 5c presents a second reversibility test focused on the halochromic switching between the merocyanine and protonated merocyanine states under repeated acid vapor exposure to HCl and TEA. Reflectance is monitored at ≈430 and ≈510 nm, corresponding to the characteristic reflectance of the merocyanine and protonated merocyanine states on AAO membranes, respectively, over seven switching cycles. In each cycle, exposure to HCl vapor induces protonation of merocyanine, resulting in a decrease in reflectance at ≈430 and 630 nm and a simultaneous increase at ≈510 nm. Subsequent exposure to TEA vapor effectively deprotonates the protonated merocyanine form, restoring the merocyanine state and reversing the optical signature. Notably, both reflectance intensities at 430 (merocyanine) and 510 nm (protonated merocyanine) exhibit a gradual and consistent decline over successive cycles. This decreasing trend reflects a progressive reduction in switching efficiency, indicating that the optical contrast weakens with repeated acid/base exposure. In contrast, the purely photo‐induced spiropyran‐to‐merocyanine cycling shown in Figure 5b displays relatively stable reflectance intensity over cycles, suggesting higher photochemical robustness of the SP‐*t*‐AAO system under light stimuli alone. The decline in reflectance intensity during the HCl/TEA cycles may be attributed to acid‐induced fatigue may occur due to repetitive protonation‐deprotonation processes at the molecular level, which can lead to chemical degradation, hydrolysis of surface linkages, or partial decomposition of the protonated chromophore.^[^ ^23^, ^65^, ^90^, ^91^ ^]^

Besides, to further quantify the reversibility of the SP‐*t*‐AAO system under repeated photochromic and halochromic switching, both morphological integrity and optical contrast are quantitatively analyzed using image‐based Canny edge detection and standard deviation (SD) metrics of pixel intensity,^[^ ^92^, ^93^ ^]^ as shown in Figure S10 (Supporting Information). Figure S10 (Supporting Information) demonstrates the edge perimeter‐preserving stability of the patterned “Spring” character on SP‐*t*‐AAO membranes during multiple UV light on/off cycles. Using Canny edge detection to track the perimeter of the photo‐induced pattern, no significant variation is observed across cycles, indicating robust retention of the spatial pattern under repeated spiropyran‐to‐merocyanine isomerization. This confirms that UV‐triggered switching does not induce morphological degradation or delamination of the grafted spiropyran layer.^[^ ^65^, ^90^, ^91^ ^]^ In parallel, Figure S10b (Supporting Information) quantifies the visual contrast over the same UV cycling sequence using the standard deviation of pixel intensity in the histogram across the patterned area. The contrast remains largely stable with only a slight decrease over time (SD from 60.7 to 50.6), supporting the conclusion that photochromic cycling exhibits high reproducibility and low fatigue.^[^ ^65^, ^90^, ^91^ ^]^ By contrast, Figure S10c,d (Supporting Information) evaluates the effect of repeated halochromic switching using HCl and TEA vapor on the same patterned “Spring” character. In Figure S10c (Supporting Information), edge detection analysis using the Canny edge detection method shows that the perimeter of the patterned “Spring” character remains clearly defined across multiple HCl/TEA vapor switching cycles. This result suggests that the covalently anchored spiropyran layer on the AAO membranes maintains spatial resolution even under repeated halochromic cycling conditions. In Figure S10d (Supporting Information), however, the contrast analyses show a significant drop in contrast as measured by SD during the same HCl/TEA cycles. Specifically, for the MC‐*t*‐AAO membrane, the standard deviation decreases from 63.6 to 22.3, corresponding to a 61.9% reduction. For the MCH‐*t*‐AAO state, the standard deviation drops from 8.1 to 5.7, reflecting a 29.6% decrease. The results highlight stronger contrast fatigue compared to UV cycling. This observation may be attributed to multiple factors, including acid‐induced hydrolysis, partial degradation of the protonated merocyanine chromophore, or accumulation of residual acid that impairs full recovery to the merocyanine state.^[^ ^23^, ^65^, ^90^, ^91^ ^]^

On the other hand, Figure 5d demonstrates a reusability test, where a single SP‐*t*‐AAO membrane is subjected to sequential cycles of exposure to different acid (TFA, HCl, CH_3_COOH, and HNO_3_) and base (TEA) vapor. It should be noted that the SP‐*t*‐AAO membrane is pre‐irradiated with high‐intensity white light for 5 min to ensure complete conversion to the colorless spiropyran form before the reusability tests, thereby eliminating any pre‐existing coloration or unintended activation caused by ambient environmental condition. The spiropyran on SP‐*t*‐AAO membrane is first activated to the merocyanine state under UV light, then exposed to one acid vapor at a time. Each acid reversibly converts the magenta merocyanine pattern to yellow protonated merocyanine, and TEA vapor restores the magenta color. This experiment highlights that the same membrane can be reused across multiple chemical (acid) environments, with obvious color switching. Notably, acids with stronger acidity or higher volatility (TFA, HCl, and HNO_3_) produce more intense yellow coloration because of more complete merocyanine protonation, while weaker acids like CH_3_COOH result in intermediate shades as a result of partial conversion. Furthermore, to quantitatively assess the reusability of the SP‐*t*‐AAO membrane under repeated photochromic and halochromic switching with different acid vapors, optical contrast analysis based on image data is performed. Figure S10e (Supporting Information) demonstrates the reusability of a single SP‐*t*‐AAO membrane subjected to sequential acid exposures (TFA, HCl, HNO_3_, and CH_3_COOH), with TEA vapor treatments between each step. The SD values obtained after each acid/base switching cycle reveal a general downward trend in optical contrast, indicating that repeated halochromic cycling, regardless of acid type, leads to progressive merocyanine fatigue.^[^ ^23^ ^]^ This decline is likely caused by partial chromophore degradation, or incomplete reversibility of the merocyanine‐to‐protonated merocyanine transformation over time.^[^ ^23^, ^65^ ^]^ Stronger acids such as TFA, HCl, and HNO_3_ initially produce high contrast because of efficient and rapid protonation but also show faster contrast decay, consistent with their higher reactivity and potential for inducing structural fatigue. Interestingly, a rebound in the contrast of MCH‐*t*‐AAO membrane is observed during the final switching step involving CH_3_COOH. Because acetic acid is weaker and less volatile, it induces only partial conversion of merocyanine to protonated merocyanine. As a result, a portion of the original reddish‐purple coloration from the merocyanine state remains within the pattern region.^[^ ^65^ ^]^ The above‐mentioned results validate that the SP‐*t*‐AAO membrane system can be a robust and versatile platform for reversible, multicycle color modulation.

**Figure 6:**
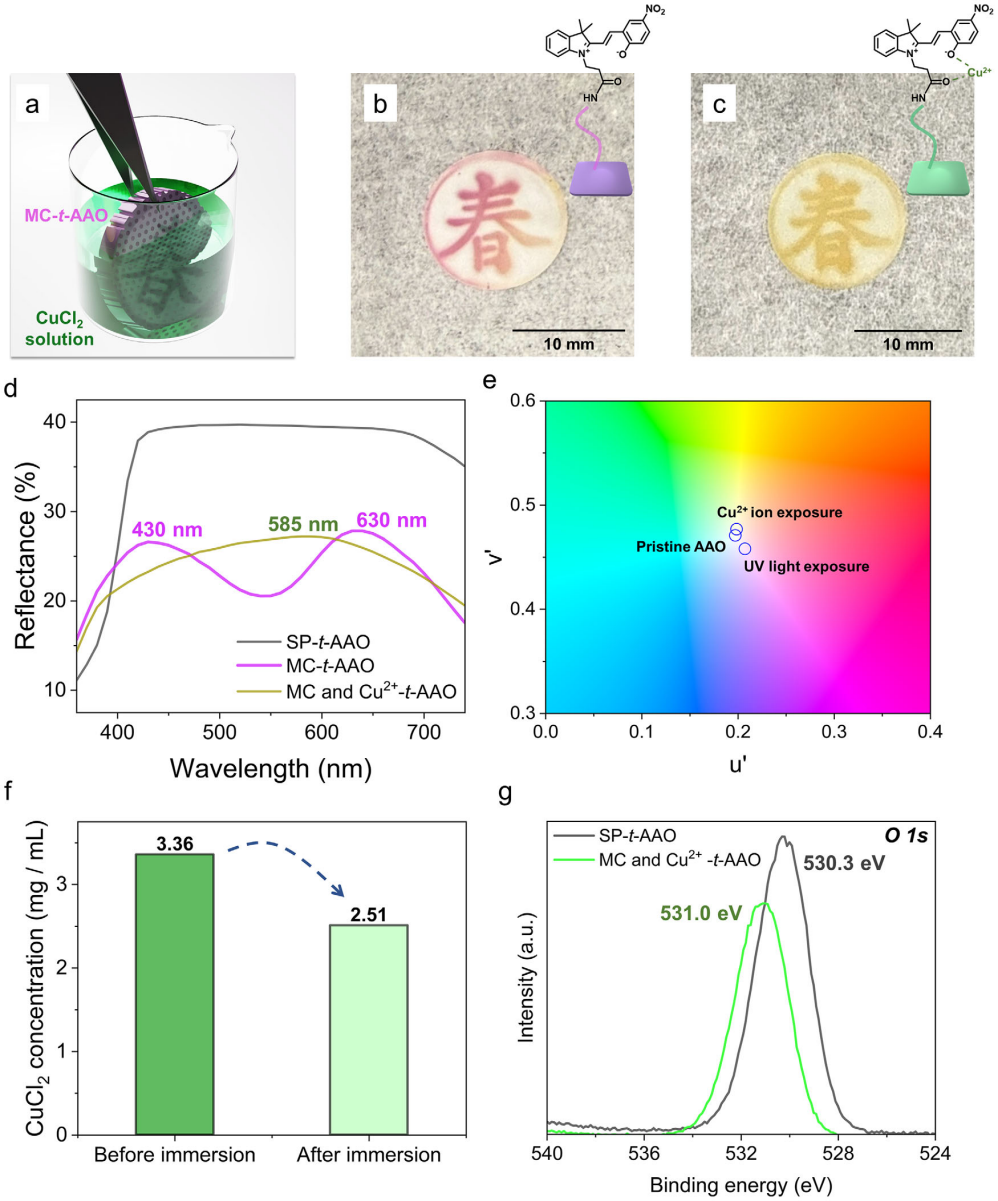
Studying the response of MC‐*t*‐AAO membranes to Cu^2+^ ion exposure. a) Schematic of MC‐*t*‐AAO membrane immersion in CuCl_2_ solution, enabling Cu^2+^‐induced chemical interaction with the merocyanine chromophore. b) Optical image of the photopatterned MC‐*t*‐AAO membrane prior to Cu^2+^ treatment, displaying a magenta “Spring” pattern. c) Image of the same membrane after Cu^2+^ ion exposure, showing a visible color shift toward yellow‐green, indicative of merocyanine‐Cu^2+^ coordination. d) Reflectance spectra showing spectral changes before and after Cu^2+^ exposure, with attenuation of the MC‐associated bands (≈430 and 630 nm) and emergence of a new band near 585 nm. e) CIE 1976 (u’, v’) chromaticity diagram illustrating the trajectory of color evolution from the pristine state to MC‐*t*‐AAO and finally to the Cu^2+^‐treated state. f) Quantitative analyses of CuCl_2_ concentration before and after MC‐*t*‐AAO membrane exposure. After removal of the membrane from the solution, which is followed by rotary evaporation, and the remaining solid mass is used to calculate the residual CuCl_2_ concentration. g) GIXPS O 1s spectra of the SP‐*t*‐AAO membrane before (gray) and after (light green) Cu^2+^ coordination.

To explore the chemical reactivity of the surface‐grafted merocyanine species toward heavy metal ions,^[^ ^23^, ^39^ ^]^ the response of the MC‐*t*‐AAO membrane to Cu^2+^ exposure is investigated,^[^ ^94^ ^]^ as shown in **Figure** **6**. Upon immersing the UV‐activated MC‐*t*‐AAO membrane into a 3.36 mg mL^−1^ CuCl_2_ ethanol solution as illustrated in Figure 6a, a distinct color change is then observed on the patterned membrane. As shown in the real image (Figure 6b), the “Spring” pattern exhibits a vivid magenta color because of the presence of the zwitterionic merocyanine form. After treatment with Cu^2+^ ions (Figure 6c), the patterned region selectively changes to a yellow‐green hue, while the surrounding unmodified AAO surface remains colorless. This spatially confined color transformation strongly supports the conclusion that Cu^2+^ ions specifically interact with the merocyanine‐modified domains, confirming the metal‐binding capability of surface‐immobilized merocyanine.^[^ ^23^, ^39^, ^94^ ^]^ As shown in the proposed coordination structure (top‐right corner of Figure 6c), Cu^2+^ binding occurs through coordination with the phenolate oxygen and the adjacent carbonyl oxygen of the amide group within the conjugated merocyanine system, forming a bidentate chelation complex.^[^ ^94^ ^]^ The underlying mechanism is attributed to ligand‐metal complexation between Cu^2^⁺ ions and the electron‐donating phenolate oxygen or carbonyl oxygen atoms of the merocyanine structure.^[^ ^23^, ^39^, ^94^ ^]^ Besides, the reflectance spectra (Figure 6d) show a decrease in the characteristic merocyanine peaks at ≈430 and ≈630 nm, accompanied by the appearance of a new band near ≈585 nm, which is indicative of the formation of a Cu^2+^‐coordinated merocyanine complex. Moreover, the chromaticity analysis using the CIE 1976 (u′, v′) diagram (Figure 6e) further confirms this shift in optical state, showing a clear trajectory from the magenta merocyanine region toward a yellow‐green tone after the Cu^2+^ ion is coordinated. It is noteworthy that this color transition occurs exclusively within the merocyanine‐patterned region, highlighting the potential of this system for spatially selective heavy metal ion sensing. To assess the extent of Cu^2+^ adsorption, the residual concentration of CuCl_2_ in the ethanol solution is quantified after the MC‐*t*‐AAO membrane is exposed to the CuCl_2_ solution. As shown in Figure 6f, the solution that has been in contact with the MC‐*t*‐AAO membrane is collected and evaporated using rotary evaporation, and the remaining solid is weighed to estimate the residual CuCl_2_ content. A significant reduction in CuCl_2_ mass is observed, further confirming the effective adsorption of Cu^2+^ ions by the MC‐*t*‐AAO membrane. In addition, GIXPS measurements of the O 1s spectra are carried out to verify the coordination interaction between Cu^2+^ and the merocyanine. As shown in Figure 6g, the O 1s peak is shifted from 530.3 eV (uncoordinated) to 531.0 eV after Cu^2+^ treatment. This positive shift is indicative of a decrease in electron density around the oxygen atoms (phenolic or carbonyl groups), which is consistent with previously reported XPS observations of Cu^2+^‐ligand complexes.^[^ ^95^ ^]^ To further evaluate the selectivity of the MC‐*t*‐AAO membrane toward a broader range of heavy metal ions, an additional experiment using Fe^2+^ ions (25 mM FeCl_2_ solution) is performed. In the extended experiment, the MC‐*t*‐AAO membrane is immersed in a 25 mM FeCl_2_ solution (Figure S11a, Supporting Information). Following UV irradiation, a well‐defined “Spring” pattern emerges in the central region of the membrane because of the generation of merocyanine moieties (Figure S11b, Supporting Information). After exposure to the FeCl_2_ solution, a pale yellow coloration is observed solely within the “Spring” patterned area, whereas the surrounding unmodified regions remain visually unchanged (Figure S11c, Supporting Information). This selective color development suggests that Fe^2+^ ions preferentially coordinate with the merocyanine‐functionalized domains. Reflectance spectra (Figure S11d, Supporting Information) exhibit a distinct peak at 570 nm, indicative of Fe^2+^ binding, while the CIE chromaticity diagram (Figure S11e, Supporting Information) shows a corresponding shift in color coordinates. The localization of these optical changes within the irradiated pattern strongly supports the selective and spatially confined chelation behavior of the MC‐*t*‐AAO membrane. Together with the previously demonstrated Cu^2+^ chelation result, these findings highlight the potential of MC‐*t*‐AAO materials for heavy metal ion sensing, selective chelation, or chemical patterning applications.

To evaluate the thermal stability of the SP‐*t*‐AAO system, the membrane is subjected to post‐UV heating treatments at increasing temperatures, and the resulting color retention is assessed both visually and quantitatively, as shown in Figure S12 (Supporting Information). Upon UV irradiation at room temperature, the MC‐*t*‐AAO membrane exhibits a vivid magenta “Spring” pattern. Subsequent thermal treatments at 100, 130, and 150 °C result in gradual fading of the magenta coloration as shown in Figure S12a (Supporting Information), indicating a temperature‐dependent decline in the optical response. This trend is partially attributed to thermally induced instability under elevated temperatures.^[^ ^23^, ^96^ ^]^ The decrease in coloration is particularly evident at 150 °C, where the photochromic contrast is visibly reduced compared to lower temperature conditions. The morphologies of patterns, however, still retain at high temperature. To quantitatively analyze this effect, pixel intensity standard deviation is calculated across the patterned region using histogram‐based image analysis (Figure S12b, Supporting Information). The data show a clear inverse correlation between temperature and visual contrast, with a reduction in standard deviation as temperature increases. This decline supports the interpretation that the merocyanine domains lose structural or chromatic integrity under thermal stress, which is likely attributed to disruption of the zwitterionic resonance structure.^[^ ^23^, ^96^ ^]^ Building upon the static thermal treatment results, thermal cycling stability is systematically investigated to assess chromic fatigue behavior under repeated thermal stress. A series of heating‐cooling‐UV light cycles is applied to the SP‐*t*‐AAO membrane, and the chromic response at each stage is monitored (Figure S13a–d, Supporting Information). In each cycle, the membrane is heated to 150 °C and held for 5 min, followed by exposure to white light and then UV light to evaluate the recovery of photoresponsiveness. Upon heating, a distinct thermochromic change is observed, which is attributed to the thermal ring‐opening isomerization of spiropyran (SP) to its merocyanine (MC) form, as reported in spiropyran‐containing systems embedded in solid matrix.^[^ ^23^, ^36^, ^97^, ^98^, ^99^, ^100^ ^]^ After UV irradiation, the patterned merocyanine form remains visible. Both the coloration and pattern contrast, however, are found to gradually fade with an increasing number of cycles. To quantify the degradation in optical performance during cycles, the standard deviation of pixel intensity is calculated from the UV‐activated images (Figure S13e, Supporting Information). A decreasing trend in pixel contrast is observed, reflecting the progressive loss of photochromic activity, likely attributed to heat‐induced fatigue and thermal fading of the spiropyran moieties.^[^ ^100^, ^101^ ^]^ Several factors may contribute to this thermal fatigue: 1) the cleavage or weakening of intramolecular or intermolecular hydrogen bonding within the spiropyran layer under elevated temperatures, which disrupts the ring‐opening reversibility;^[^ ^100^ ^]^ 2) partial thermal oxidation or decomposition of the surface‐bound organic groups, as supported by the thermogravimetric analysis (TGA, Figure S13e, Supporting Information), which reveals an onset of weight loss above ≈100 °C; 3) molecular aggregation of the MC form upon thermal activation, leading to reduced reversibility because of stabilization of the open‐ring configuration and suppression of spiropyran;^[^ ^100^, ^101^ ^]^ and 4) irreversible surface compositional changes, as evidenced by GIXPS (Table S1, Supporting Information), showing decreased C, N, and S contents and increased O content after thermal cycling, indicating partial degradation or desorption of the spiropyran layer. Collectively, the results conclude that while the SP‐*t*‐AAO system retains partial chromic responsiveness after repeated thermal exposure, some decline in performance is observed under elevated temperatures. These findings highlight the importance of further studies on long‐term thermo‐oxidative aging in ambient conditions and extended thermal cycling, which will be valuable for thoroughly assessing the material's potential in high‐temperature or aerospace‐related applications.

The long‐term stability is crucial for practical applications such as information storage and anti‐counterfeiting. To evaluate this, the chromogenic stability of the SP‐*t*‐AAO membranes is investigated under ambient storage conditions for 1, 10, and 20 days. As shown in Figure S14a (Supporting Information), prior to each measurement, the samples are irradiated with UV light to trigger the photochromic transition from spiropyran to its colored merocyanine form. The magenta‐colored patterns are observed to remain clearly visible with no significant fading over time. In addition, the image‐based contrast is quantitatively assessed by calculating the standard deviation (SD) of the histogram within the patterned regions (Figure S14b, Supporting Information). The SD values are found to remain consistent, from 41.42 (Day 1) to 41.01 (Day 10) and 44.02 (Day 20), indicating that the SP‐*t*‐AAO membranes retain reliable optical contrast and chromogenic durability over extended storage durations. Besides, to further assess the environmental robustness of the SP‑*t*‑AAO membrane, additional tests simulating humidity exposure and mechanical abrasion are conducted. As shown in Figure S15a (Supporting Information), the membrane is suspended above boiling water to generate saturated water vapor. After prolonged exposure, the membrane surface shows no apparent change, indicating that the presence of moisture alone does not induce ring‐opening isomerization of spiropyran (Figure S15c, Supporting Information). Upon the subsequent UV irradiation, the membrane still exhibits a vivid and high‐contrast “Spring” character (Figure S15d, Supporting Information), confirming that its photochromic functionality remains intact even after humidity treatment. In addition, the membrane's mechanical durability is also evaluated using an abrasion test with CW 1000 sandpaper, as illustrated in Figure S16 (Supporting Information). After photoactivation to the MC‑*t*‑AAO state, the membrane is subjected to repeated surface scratching. While the pattern remains visible, its sharpness and definition are noticeably reduced after the abrasion. The results suggest that although the membrane retains its optical switching capability, its resistance to physical wear is limited, likely attributed to the inherent brittleness of the anodized aluminum oxide substrate.

The comparison is also important for evaluating the practical performance of anti‐counterfeiting systems. Therefore, the comparisons highlighting key performance metrics, including color contrast (ΔE), response time, and cyclic durability are carried out, as shown in Table S2 (Supporting Information). The system in this work achieves high color contrast values of 28.99 (photochromism) and 27.02 (halochromism), ensuring visual distinguishability. The SP‐*t*‐AAO membrane also exhibits rapid switching behavior, with acid‐induced responses occurring within 0.4–4 s and photo‐induced responses completing within 60 seconds. In terms of durability, our platform supports over 10 reversible photochromic cycles and maintains moderate performance under repeated halochromic stimuli. Importantly, the integration of dual‐stimuli responsiveness, light and acid/base, enables orthogonal and independent activation modes, offering greater flexibility and security than conventional single‐mode systems. These advantages collectively demonstrate the competitiveness and applicability of our system in advanced anti‐counterfeiting applications. Given its balanced performance in all three aspects, color contrast, response time, and switching durability, the SP‐*t*‐AAO membrane holds strong potential for real‐world applications in areas requiring security, such as document security and smart labeling technologies.

## 3 Conclusions

In summary, this study presents a comprehensive strategy for designing SP‐*t*‐AAO membranes with spatially controlled photo‐responsive properties. By employing sequential thiol‐yne click chemistry and photopatterning using Chinese seasonal‐themed photomasks, spiropyran molecules are selectively grafted onto amine‐functionalized regions of the AAO surface, enabling reversible color switching upon exposure to UV light (photochromism) and acidic/basic vapors (halochromism). The success of this orthogonal surface modification is verified by GIXPS, EDS, and TOF‐SIMS analyses, which confirm its spatial resolution, chemical selectivity, and uniform modification across micrometer‐scale depths. Quantitative spectroscopic and image‐based analyses demonstrate excellent photochromic reversibility and moderate halochromic fatigue over 10 switching cycles, with color contrast loss attributed to spiropyran/merocyanine degradation or incomplete reversibility. High‐frame‐rate video analyses (0.01 s intervals) further confirms the fast halochromic response (0.4–4 s), dependent on acid strength and vapor pressure. Additionally, merocyanine‐modified membranes exhibit spatially selective reactivity with both Cu^2+^and Fe^2+^ ions, with metal‐chelation localized within the patterned areas as confirmed by colorimetric mapping, GIXPS and reflectance spectroscopy, reinforcing the potential of SP‐*t*‐AAO for molecular recognition and heavy metal ion sensing. Thermal stability tests reveal that the patterned morphology is retained at elevated temperatures, although chromogenic contrast gradually fades during thermal cycling because of chromophore decomposition, as evidenced by TGA analysis. Moreover, environmental and mechanical robustness evaluations indicate that the membrane maintains its switching function under water vapor exposure and mild abrasion. The long‐term mechanical durability, however, remains limited by the inherent brittleness and mechanical hardness of the AAO membrane. Collectively, these findings establish SP‐*t*‐AAO membranes as a tunable, reusable, and spatially defined dual‐stimuli platform with promising applications in sensing, anti‐counterfeiting, and information encryption materials.

## 4 Experimental Section

### Materials

Ethanol (>99.8%), acetic acid (ACS reagent, 99.8%), hydrochloric acid (ACS reagent, fuming, ≥37%), and hydrogen peroxide (35 wt.%) were purchased from Honeywell. Anodic aluminum oxide (AAO) membranes were acquired from Whatman. Anhydrous diethyl ether (99%), acetone (99.5%), and ethanol (99.5%) were obtained from Echo. 5‐Nitrosalicylaldehyde (98%), *1H,1H,2H,2H*‐perfluorodecanethiol (98%), and cysteamine hydrochloride (99%) were acquired from Nova Materials. 2,2‐Dimethoxy‐2‐phenylacetophenone was obtained from Sigma–Aldrich. 10‐Undecynylphosphonic acid (>95%) was acquired from SIKÉMIA. The 12 mm seasonal‐themed photomask mask, which was made of stainless steel, was obtained from MicroMetal Technology. 3‐Iodopropionic acid (99%), triethylamine (TEA, 99.0%), and copper(II) chloride (anhydrous, 98%) were obtained from Alfa Aesar. *N*‐hydroxysuccinimide (NHS, 98%), dimethylformamide (99.8%), and iron(II) chroride (97%, anhydrous) were purchased from Acros. 1‐(3‐Dimethylaminopropyl)‐3‐ethylcarbodiimide hydrochloride (EDC, 95%) was obtained from Matrix. Toluene (ACS reagent, 99.5%) and nitric acid (69.0‐70.0%) were obtained from J.T. Bakers. Trifluoroacetic acid (TFA) was supplied from Thermo Scientific.

### Synthesis of Spiropyran Containing Carboxylic Acids (SP‐COOH)

SP‐COOH was synthesized according to a previously reported procedure.^[^ ^65^, ^68^ ^]^ Initially, 2,3,3‐trimethylindolenine (4.0 g, 25 mmol) and 3‐iodopropionic acid (5.4 g, 27 mmol) were dissolved in 10 mL of toluene and refluxed under nitrogen‐purged conditions for 12 h. Upon completion, the reaction mixture was cooled to room temperature and filtered. The resulting iodide salt was washed thoroughly with cold diethyl ether and subsequently dried under vacuum. In the subsequent step, the iodide salt (4.5 g, 12.6 mmol), 5‐nitrosalicylaldehyde (2.1 g, 12.6 mmol), and piperidine (1.3 mL, 13.2 mmol) were mixed in a round‐bottom flask. The mixture was refluxed for 5 h and then stirred at room temperature for an additional 5 h. After cooling to 0 °C in a fridge, the reaction mixture yielded a yellow green precipitate. The crude product was collected by gravity filtration and washed several times with cold ethanol to afford the desired SP‐COOH compound.

### Fabrication of 10‐Undecynyl‐*t*‐AAO Membrane

An AAO membrane was first immersed in a sample bottle containing 35 wt.% hydrogen peroxide and heated on a hotplate at 100 °C for 1 h. After this treatment, the membrane was transferred to a plasma chamber. Once the pressure in the chamber dropped below 200 mTorr, oxygen gas was supplied, and the membrane was exposed to O_2_ plasma at 180–200 V for 5 min. Following plasma treatment and the return to ambient pressure, the membrane‐now processed using both H_2_O_2_ solution and O_2_ plasma—was prepared for surface functionalization. A solution of 10‐undecynylphosphonic acid (0.4 mg mL^−1^ in anhydrous ethanol) was prepared, and the treated AAO membrane was immersed in the solution on a hotplate at 50 °C for 12 h. Afterward, the membrane was removed and thoroughly rinsed with ethanol and acetone. Finally, the membrane was dried under vacuum, yielding the 10‐undecynyl‐terminated AAO (10‐undecynyl‐*t*‐AAO) membrane.

### Fabrication of the Selective Thiol‐*t*‐AAO Membrane

A solution containing 15 wt.% cysteamine hydrochloride and 1.0 wt.% 2,2‐dimethoxy‐2‐phenylacetophenone in DMF was first prepared and drop‐cast onto the 10‐undecynyl‐*t*‐AAO membrane. A seasonal‐themed photomask (spring, fall, summer, and winter) was placed on the membrane, and the sample was then exposed to 254 nm UV light (4 Watt) for 5 min. After the exposure, the membrane was thoroughly rinsed with ethanol to remove unreacted components. Next, a solution of 15 wt.% *1H,1H,2H,2H*‐perfluorodecanethiol with 1.0 wt.% 2,2‐dimethoxy‐2‐phenylacetophenone in DMF was deposited on the partially modified membrane. The sample was again exposed to 254 nm UV light (4 Watt) for 5 min and then washed with ethanol. This process yielded the selective thiol‐*t*‐AAO membrane. Alternatively, the sequence of modification can be reversed. The AAO membrane can first be modified with *1H,1H,2H,2H*‐perfluorodecanethiol under UV irradiation, followed by the deposition of cysteamine hydrochloride for further photochemical grafting. This reversed process allows for the creation of a selective thiol‐*t*‐AAO membrane with opposite surface chemical properties.

### Fabrication of a SP‐*t*‐AAO Membrane for Anti‐Counterfeiting Applications

SP‐COOH (10 mg) was first dissolved in 10 mL of ethanol. Subsequently, 30 mg of *N*‐hydroxysuccinimide (NHS) was added to the solution and stirred until fully dissolved. Next, 50 mg of 1‐ethyl‐3‐(3‐dimethylaminopropyl)carbodiimide (EDC) was introduced to activate the carboxyl groups. A selectively thiol‐*t*‐AAO membrane featuring a seasonal‐themed pattern was then immersed in the reaction mixture and maintained at room temperature for 24 h. Afterward, the membrane was washed three times with ethanol using the ultrasonic cleaner and then dried under vacuum, yielding the SP‐*t*‐AAO membrane for anti‐counterfeiting applications.

### Reversible Photochromic Test of SP‐*t*‐AAO Membranes for Anti‐Counterfeiting Display

An SP‐*t*‐AAO membrane was initially exposed to a 365 nm UV light source for 1 min, resulting in the formation of the MC‐*t*‐AAO membrane. This photoinduced transformation is reversible; the MC‐*t*‐AAO membrane gradually returned to its original SP‐*t*‐AAO state after being exposed to white light within 1 min.

### Acid / Base Responsiveness, Reversibility, and Reusability of MC‐*t*‐AAO Membranes for Anti‐Counterfeiting Exhibitions

Different acid solutions‐trifluoroacetic acid (TFA), hydrochloric acid (HCl), nitric acid (HNO_3_), and acetic acid (CH_3_COOH)‐as well as a base solution, triethylamine (TEA), were individually prepared in separate sample bottles. The MC‐*t*‐AAO membranes were exposed to each of the four acid vapors for 5 s to form the corresponding protonated MCH‐*t*‐AAO membranes. These membranes were then reverted to their original MC‐*t*‐AAO state upon exposure to TEA vapor for 5 s. For reversibility tests, concentrated hydrochloric acid and TEA were used as representative acid and base sources, respectively. In the reusability test, the MC‐*t*‐AAO membranes were sequentially exposed to the four acid vapors‐TFA, HCl, HNO_3_, and CH_3_COOH‐with TEA vapor treatment applied between each acid exposure to regenerate the MC‐*t*‐AAO state before the next cycle.

### Cu^2+^ Absorption Test of MC‐*t*‐AAO Membranes for Anti‐Counterfeiting Demonstration

A 10 mL ethanol solution of CuCl_2_ (25 mM) was first prepared in a sample bottle. The MC‐*t*‐AAO membrane was then immersed in the solution for 5 min. After removal, the membrane was rinsed with ethanol and dried under vacuum. The remaining CuCl_2_ solution was subsequently evaporated using a rotary evaporator, and the amount of Cu^2+^ absorbed by the MC‐*t*‐AAO membrane was determined by calculating the difference in CuCl_2_ concentration before and after immersion.

### Fe^2+^ Absorption Test of MC‐*t*‐AAO Membranes for Anti‐Counterfeiting Demonstration

A 10 mL ethanol solution of FeCl_2_ (25 mM) was first prepared in a sample bottle. The MC‐*t*‐AAO membrane was then immersed in the solution for 5 min. After removal, the membrane was rinsed with ethanol and dried under vacuum.

### Analysis and Characterization

Grazing incidence X‐ray photoelectron spectroscope (GIXPS, ULVAC‐PHI PHI QuanteraII) with a grazing angle of 15° was used for surface chemical analysis of the AAO membranes after consecutive chemical modifications and metal ion chelation by the MC‐*t*‐AAO membrane. Energy‐dispersive X‐ray spectroscope (EDS, Oxford EDS 7585) was employed to analyze the elemental composition of the fluorine‐functionalized AAO (fluorine‐*t*‐AAO) membrane. Thermal stability of the spiropyran‐functionalized AAO (SP‐*t*‐AAO) membrane was evaluated using thermogravimetric analyzer (TGA55, TA Instruments). Water contact angle measurements of AAO membranes and those after chemical modifications were carried out using a contact angle goniometer (FTA125, First Ten Ångstroms) with a CCD camera. The contrast and Canny edge analyses of images were characterized by ImageJ software. ^1^H NMR spectrum of SP‐COOH was acquired by an NMR spectrometer (JEOL JNM‐ECZ400S/L1). Electrospray ionization mass (ESI‐MS) spectra of SP‐COOH were obtained by a mass spectrometer (Bruker, Impact HD, EVOQ). UV–vis absorption spectra were obtained from 300 to 700 nm using a Hitachi U‐4100 spectrometer. Reflectance spectra of AAO membranes were recorded using a Konica Minolta CM‐5 spectrophotometer, spanning a wavelength range of 360 to 740 nm. Surface molecular analyses of the chemically modified AAO membranes were performed using time‐of‐flight secondary ion mass spectrometry (TOF‐SIMS, ION‐TOF, TOF‐SIMS V) equipped with a Bi⁺ primary ion source.

## Conflict of Interest

The authors declare no conflict of interest.

## Supporting information



Supporting Information

## Data Availability

The data that support the findings of this study are available from the corresponding author upon reasonable request.
