# Supplementary material for: Dual‐Stimuli Chromogenic Membranes for Optical Security: Photochromic and Halochromic Anti‐Counterfeiting Applications
Source: Small. 2025 Aug 4;21(37):e07008. doi: 10.1002/smll.202507008 (PMC12444903; doi:10.1002/smll.202507008)
Supplement: Supplementary file 1 — Supporting Information [file SMLL-21-e07008-s001.docx]

**Supporting Information**

Dual-Stimuli Chromogenic Membranes for Optical Security: Photochromic and Halochromic Anti-Counterfeiting Applications

Lin-Ruei Lee,^1^ Yi-Fan Chen,^1^ Po-Xin Fan,^1^ Yu-Chun Lin,^1^ Ming-Hsuan Chang,^1^ Yu-Chun Liu,^1^ Chun-Chi Chang,^1^ and Jiun-Tai Chen^*12^
^1.^Department of Applied Chemistry, National Yang Ming Chiao Tung University, Hsinchu, Taiwan 300093

^2.^Center for Emergent Functional Matter Science, National Yang Ming Chiao Tung University, Hsinchu, Taiwan 300093

*To whom correspondence should be addressed. E-mail: jtchen@nycu.edu.tw. Tel.: +886-3-5731631

**
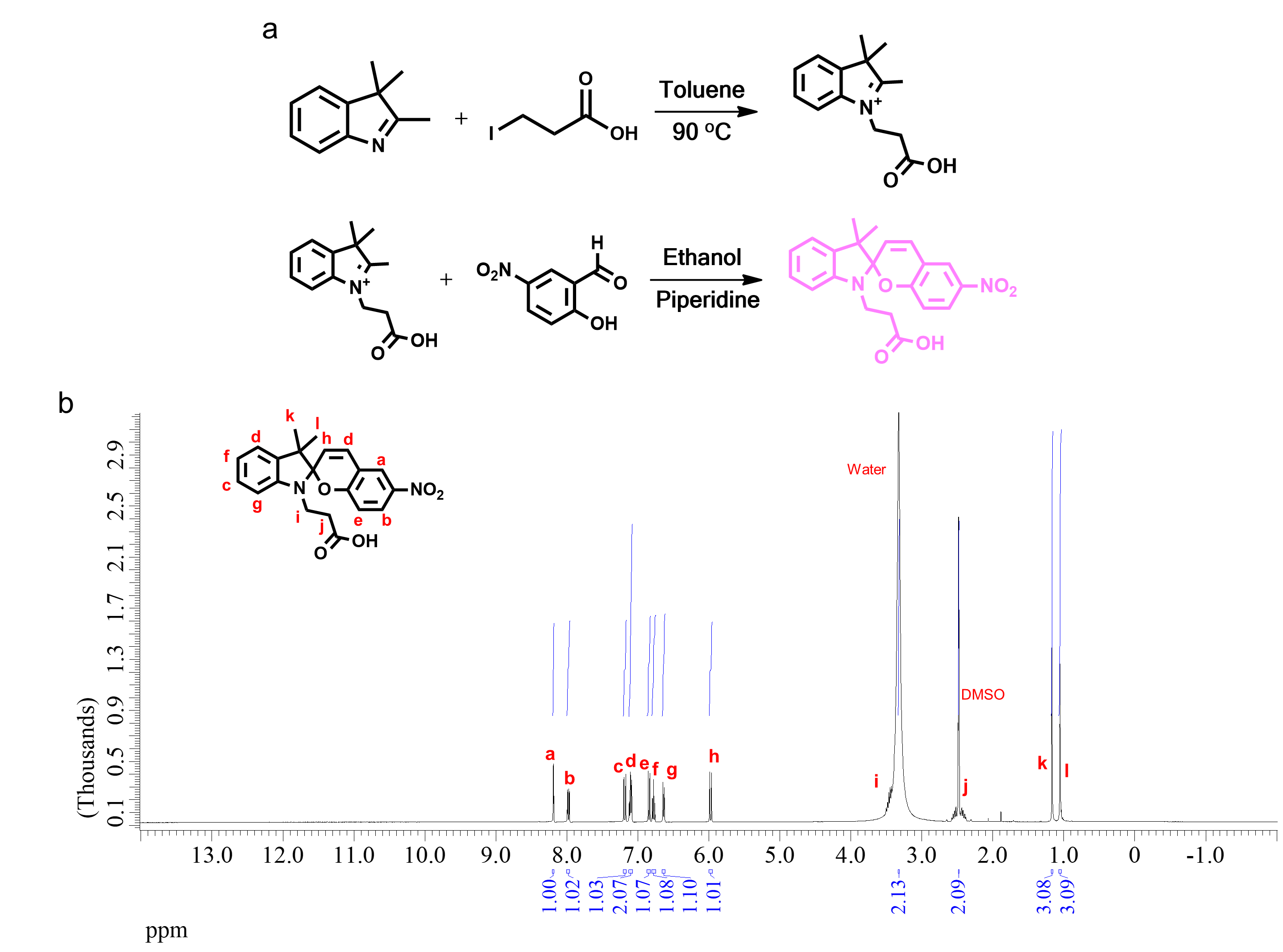
**

**Figure S1.** (a) Schematic representation of SP-COOH synthesis. (b) ^1^H NMR spectrum of SP-COOH.

_
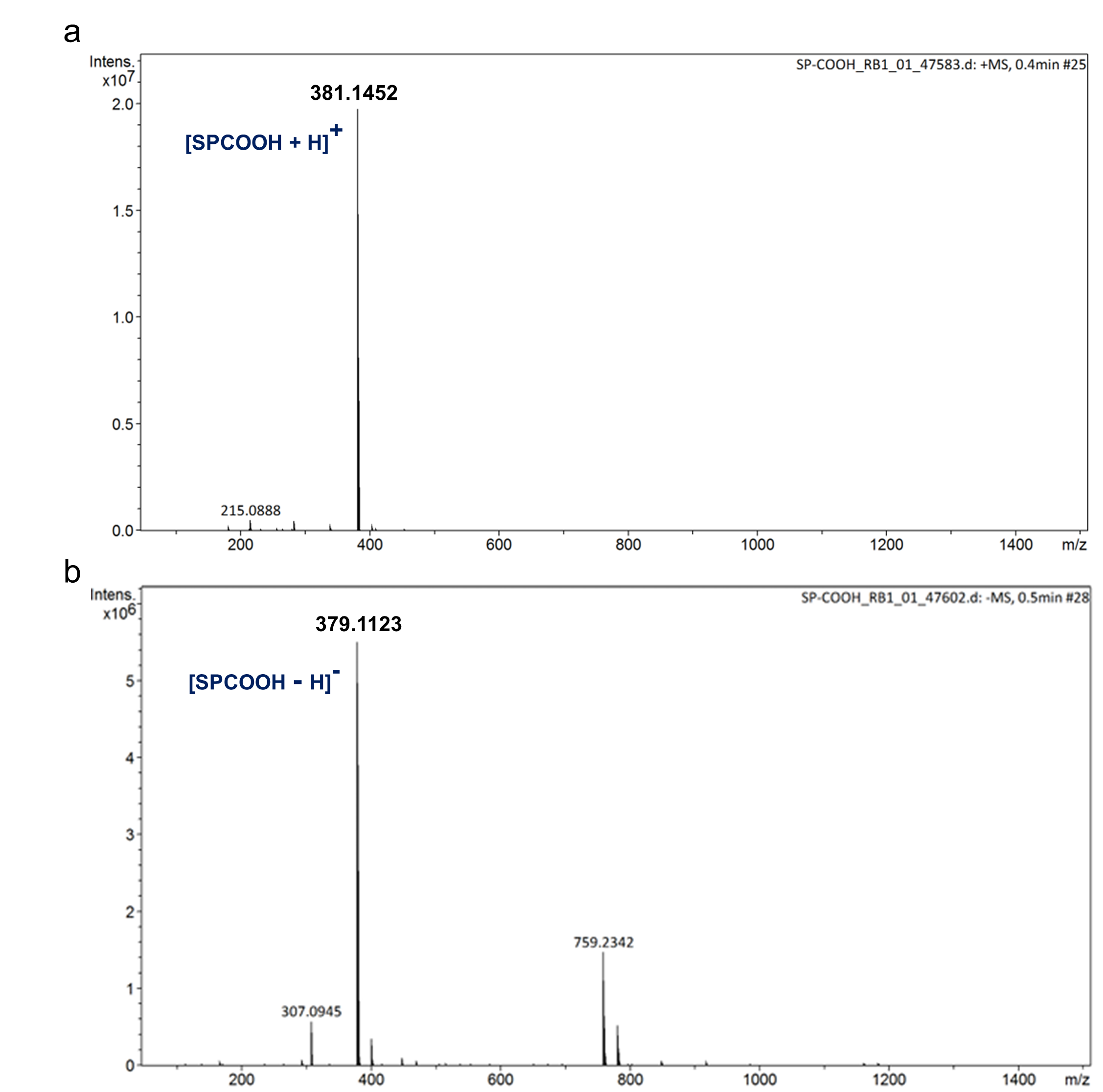
_

**Figure S2.** ESI-MS spectra of SP-COOH: (a) positive ion mode spectrum showing the [SP-COOH + H]^+^ peak at m/z = 381.1452 and (b) negative ion mode spectrum showing the [SP-COOH – H]^–^ peak at m/z = 379.1123.

_
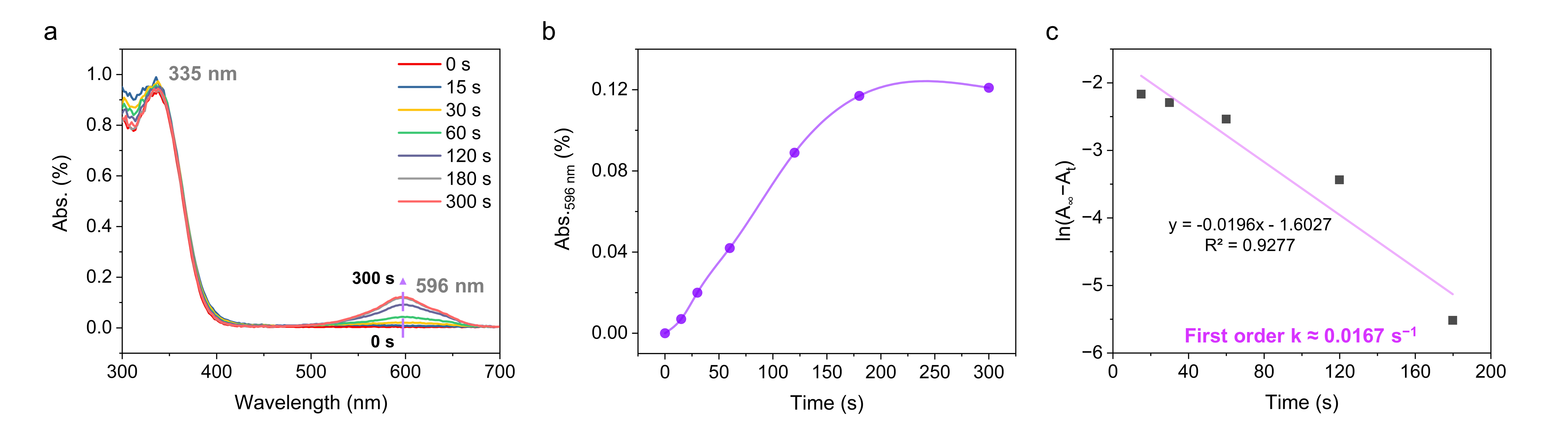
_

**Figure S3.** (a) UV-Vis absorption spectra of SP-COOH in solution under UV irradiation for varying durations (0-300 s). The initial peak at ~335 nm corresponds to SP-COOH, while the emergence of the peak at ~596 nm indicates the formation of MC-COOH through a photoinduced ring-opening reaction. (b) Time-dependent increase in absorbance at ~596 nm, indicating the progressive formation of MC-COOH. (c) First-order kinetic fitting of the ring-opening reaction from SP-COOH to MC-COOH based on the natural logarithm of the difference between final and measured absorbance values.


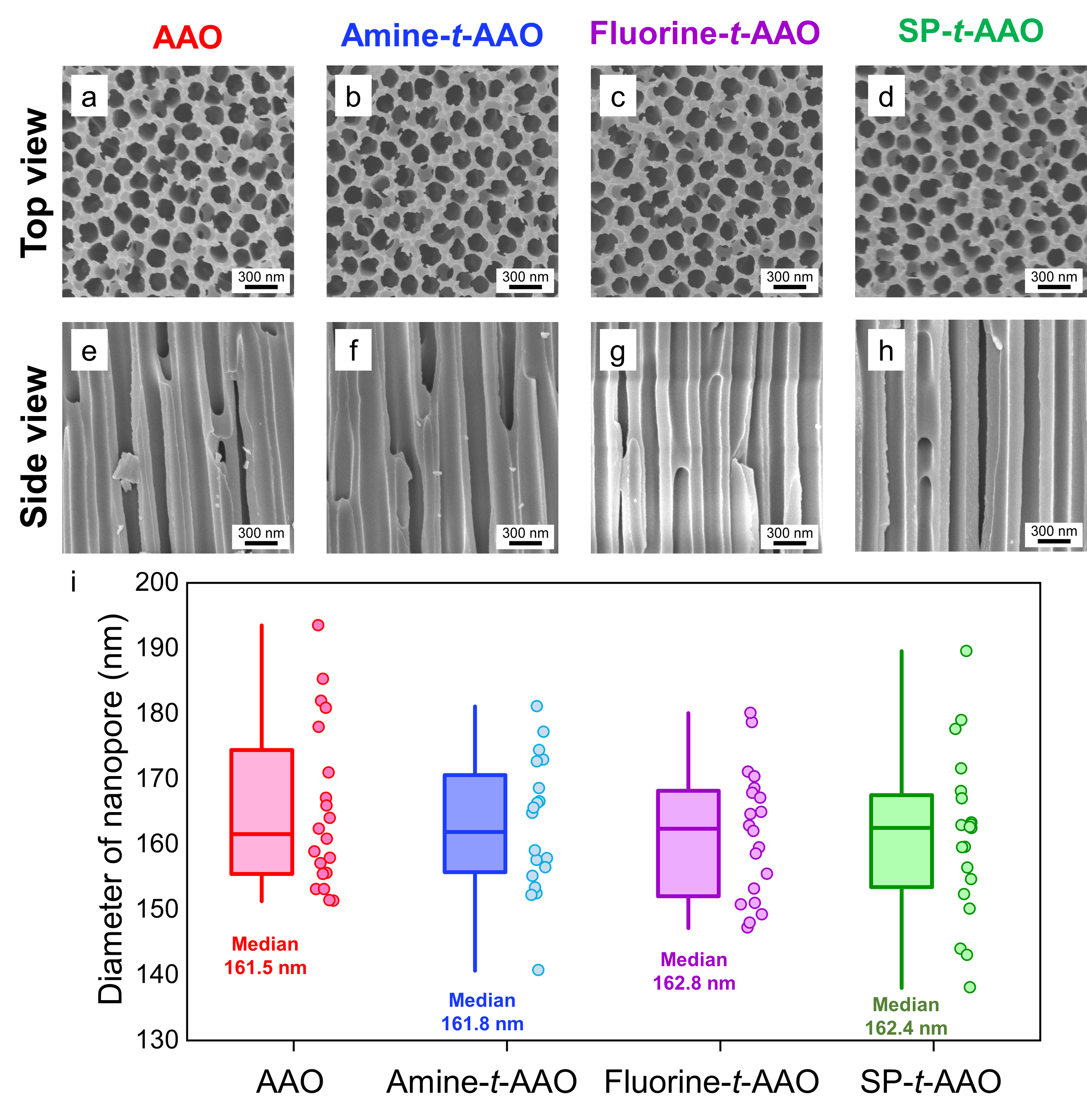


**Figure S4.** Top-view (a-d) and cross-sectional (e-h) SEM images of pristine AAO (a, e), Amine-*t*-AAO (b, f), Fluorine-*t*-AAO (c, g), and SP-*t*-AAO (d, h) membranes. (i) Statistical analyses (box plots) showing pore diameters of the AAO membranes before and after serial modifications with median values of 161.5-162.8 nm.

_
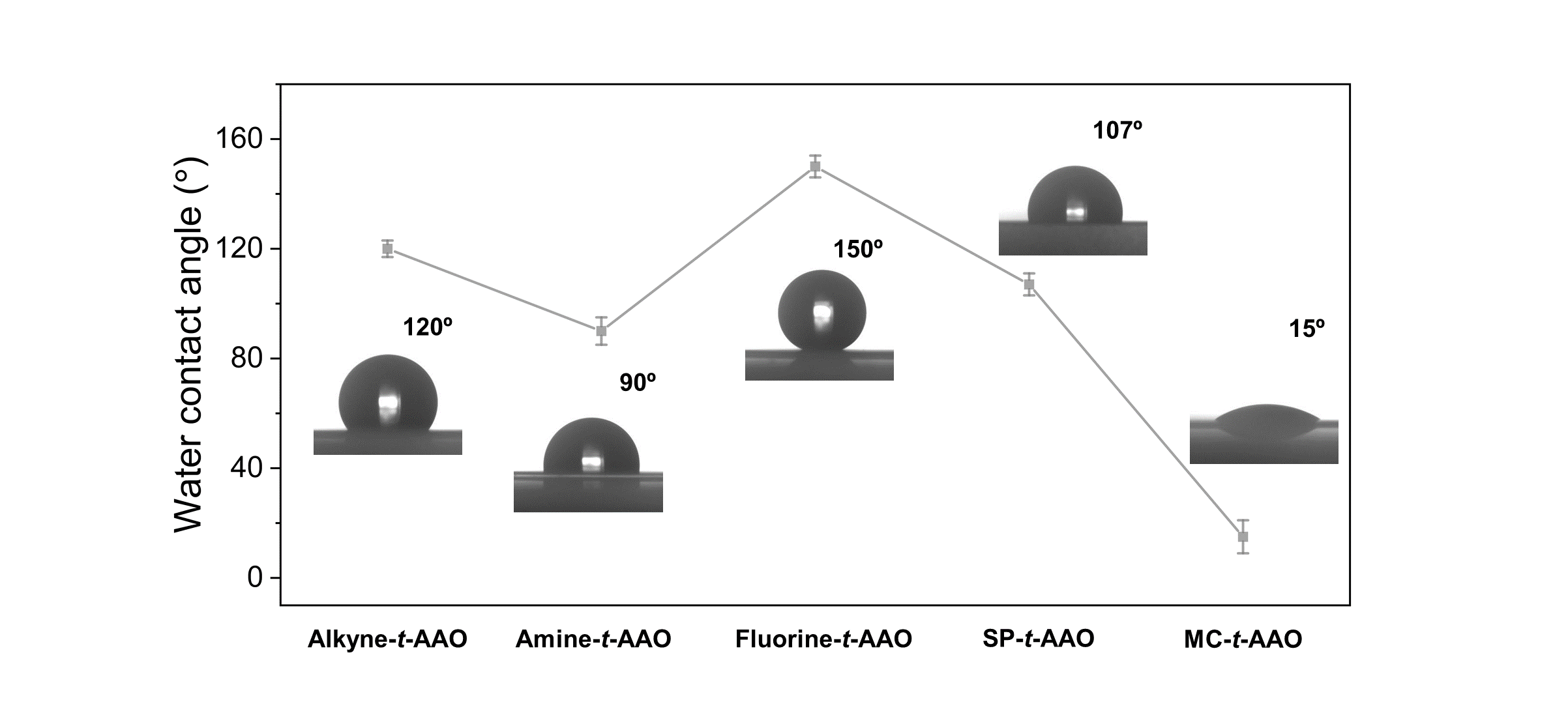
_

**Figure S5.** Static water contact angles of AAO membranes after introducing various functional groups: alkyne (alkyne-*t*-AAO), amine thiol (amine-*t*-AAO), fluorine thiol (fluorine-*t*-AAO), spiropyran (SP-*t*-AAO), and UV-induced merocyanine (MC-*t*-AAO).


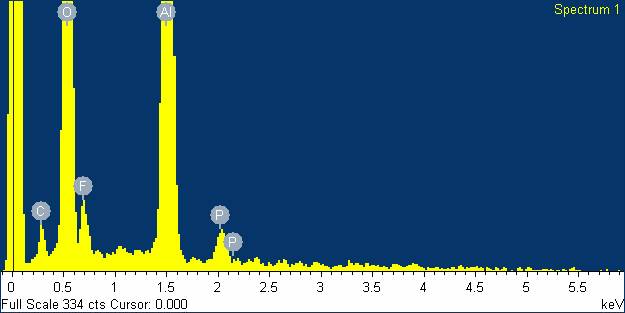


**Figure S6.** EDS spectrum of the fluorine-*t*-AAO membrane.


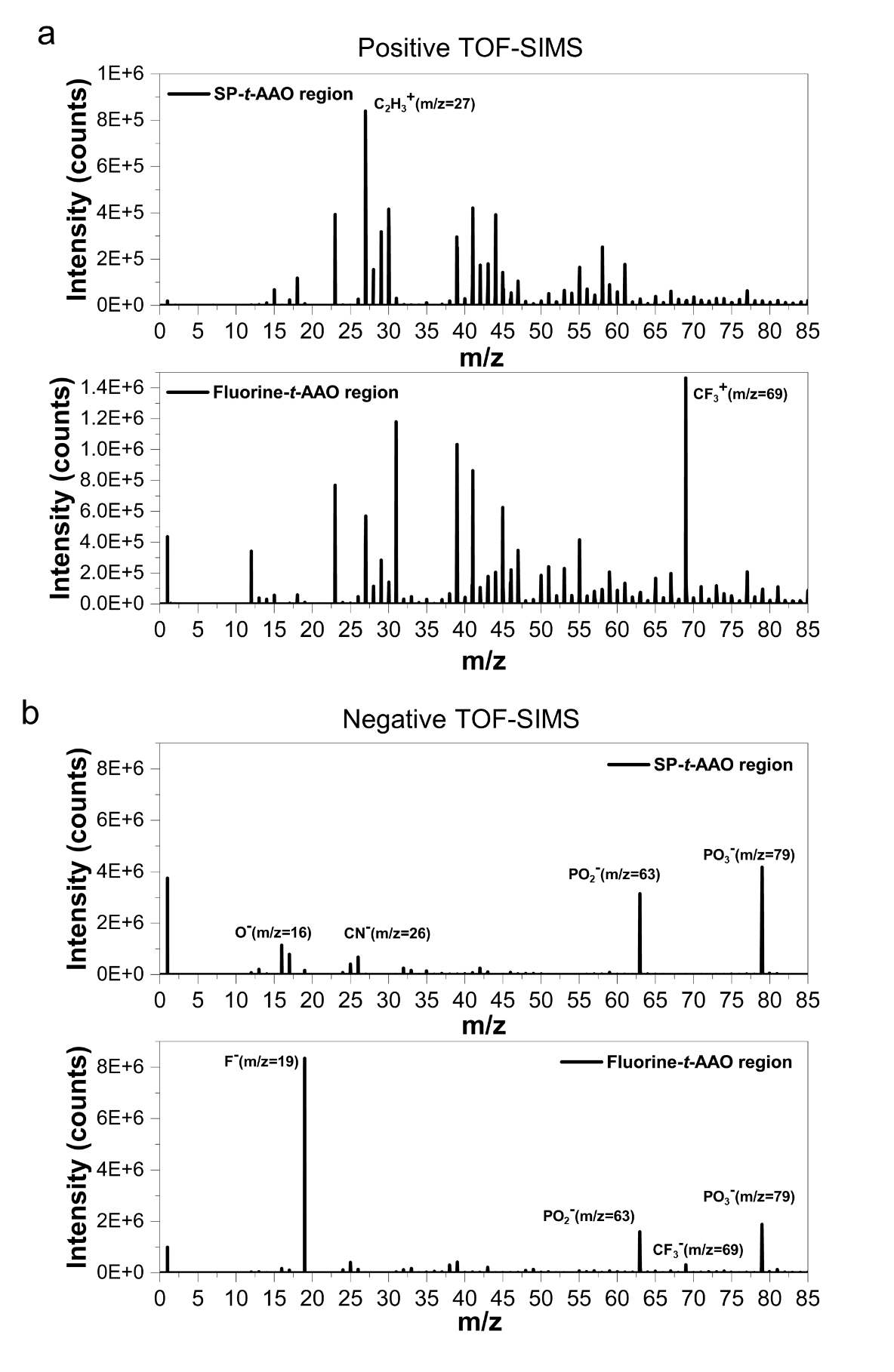


**Figure S7.** (a) Positive ion mode TOF-SIMS spectra of a SP-*t*-AAO region (top) and a fluorine-*t*-AAO region (bottom). (b) Negative ion mode TOF-SIMS spectra of a SP-*t*-AAO region (top) and a fluorine-*t*-AAO region (bottom).


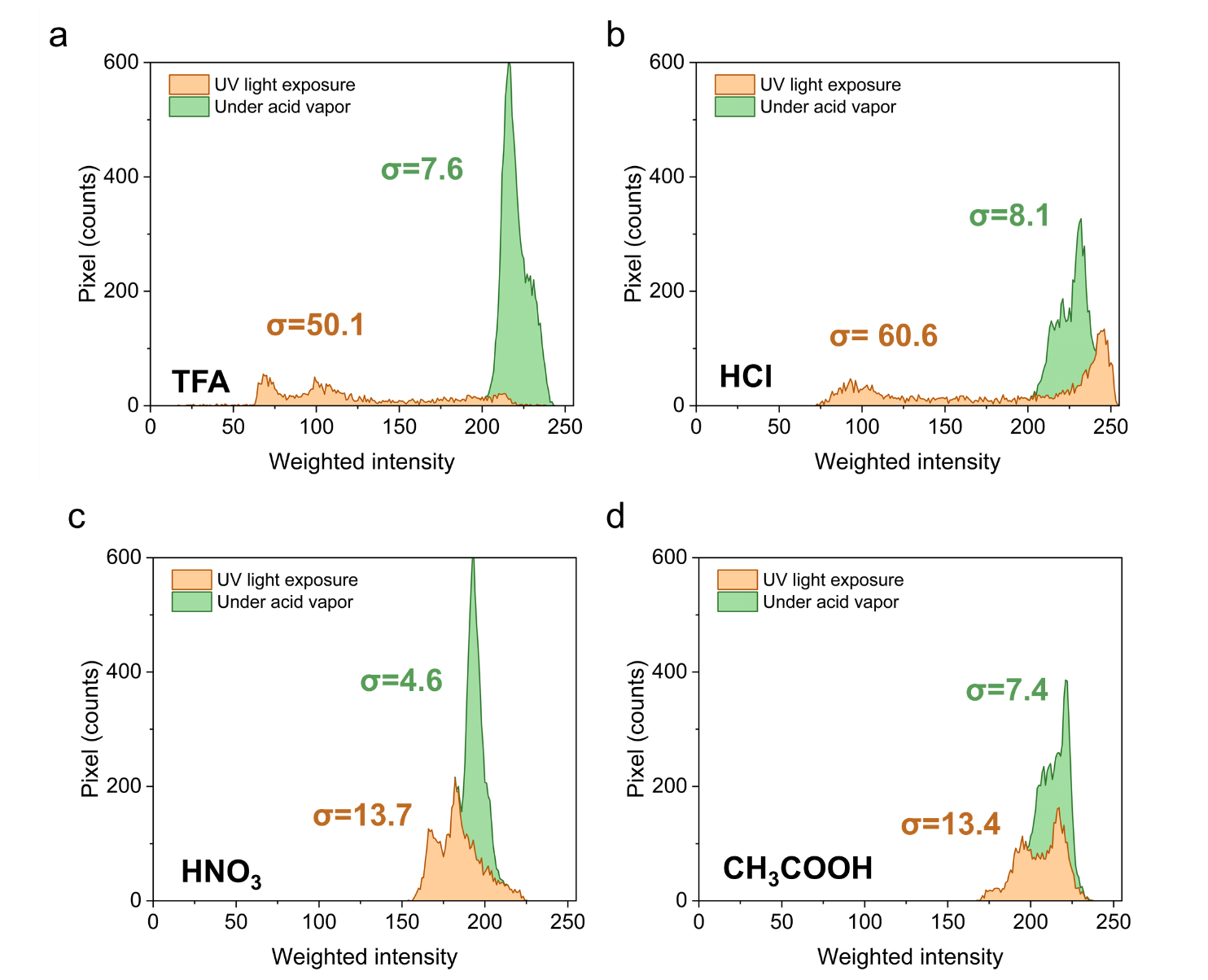


**Figure S8.** Pixel intensity histograms of SP-*t*-AAO membranes after UV light exposure (orange) and subsequent acid vapor treatment (green), used to quantify optical contrast and uniformity of color change: (a) TFA, (b) HCl, (c) HNO_3_, and (d) CH_3_COOH treatments.


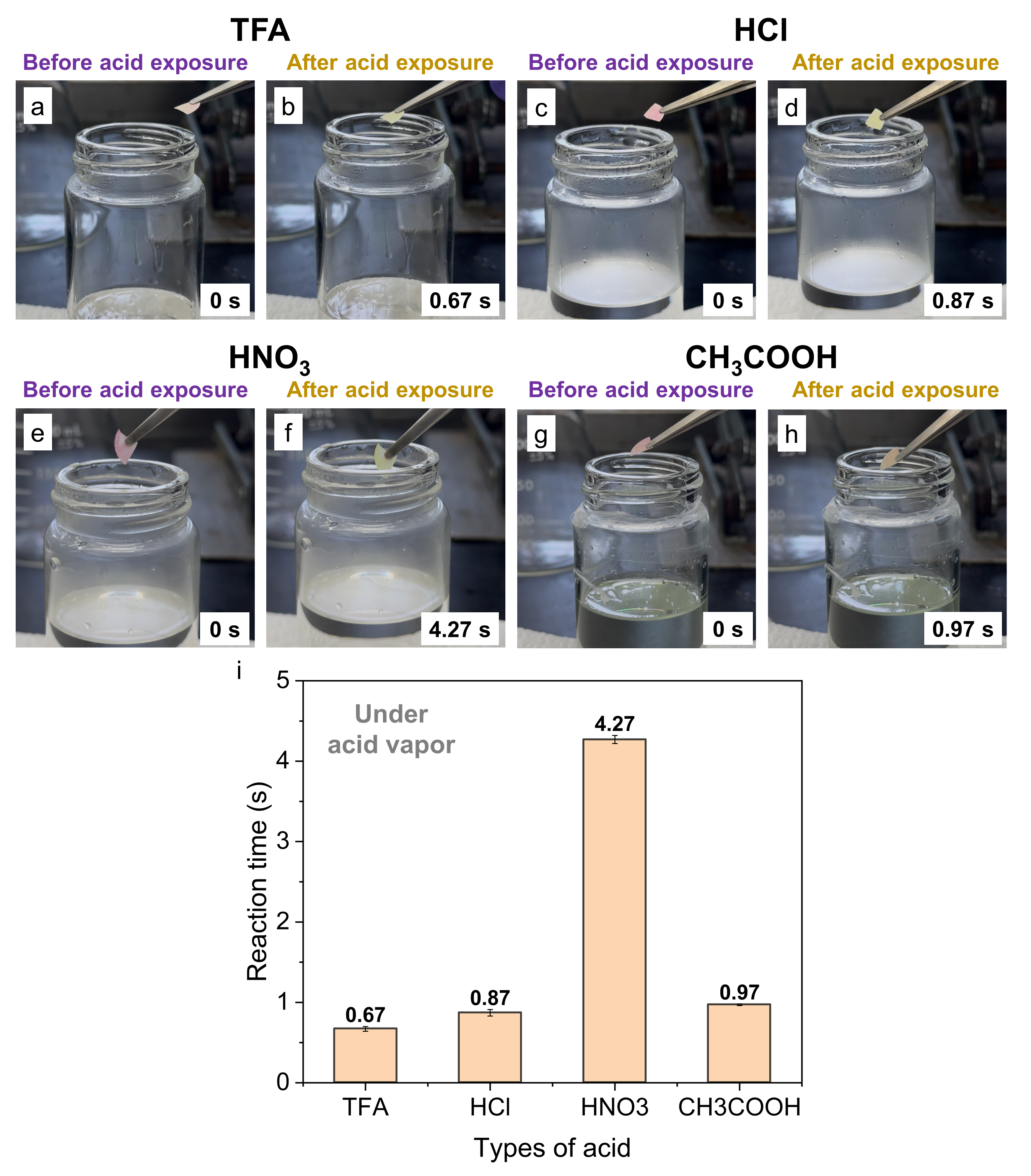


**Figure S9.** Time-resolved visual halochromic responses of MC-*t*-AAO membranes under exposure to different acid vapors. Color changes from purple to yellow: (a-b) under TFA vapor (0.67 s),(c-d) under HCl vapor (0.87 s), (e-f) under HNO_3_ vapor (4.27 s), and (g-h) under CH_3_COOH vapor (0.97 s). Images are extracted from video recordings at 0.01 s intervals to determine the time required for full visible color transition from merocyanine to protonated merocyanine form. (i) Bar chart summarizing the response times under different acid vapors.

**Note S1. Chromaticity coordinate calculation process from reflectance spectra**

The calculation is performed using the CIE 1964 10° standard observer and D65 illuminant, which are recommended for evaluating large-area color responses typical of surface-functionalized membranes. Tristimulus values X, Y, and Z are obtained by integrating the reflectance spectrum R(λ), weighted by both the spectral power distribution of the illuminant I(λ) and the standard observer’s color matching functions x̄(λ), ȳ(λ), and z̄(λ), over the visible spectrum (360-740 nm):

X = (1/N) ∫ R(λ)·I(λ)·x̄(λ) dλ
Y = (1/N) ∫ R(λ)·I(λ)·ȳ(λ) dλ
Z = (1/N) ∫ R(λ)·I(λ)·z̄(λ) dλ

where the normalization constant N ensures photometric consistency and is defined as:

N = ∫ I(λ)·ȳ(λ) dλ

These tristimulus values are then used to calculate the CIE 1976 chromaticity coordinates (u′, v′), which more uniformly represent perceptual differences in color than earlier chromaticity systems:

u′ = 4X / (X + 15Y + 3Z)
v′ = 9Y / (X + 15Y + 3Z)

This method accurately translates the spectral reflectance behavior of non-emissive materials into quantitative colorimetric values. All calculations are carried out using the “Chromaticity Diagram Plugin” developed by OriginLab Technical Support in OriginPro 2024.


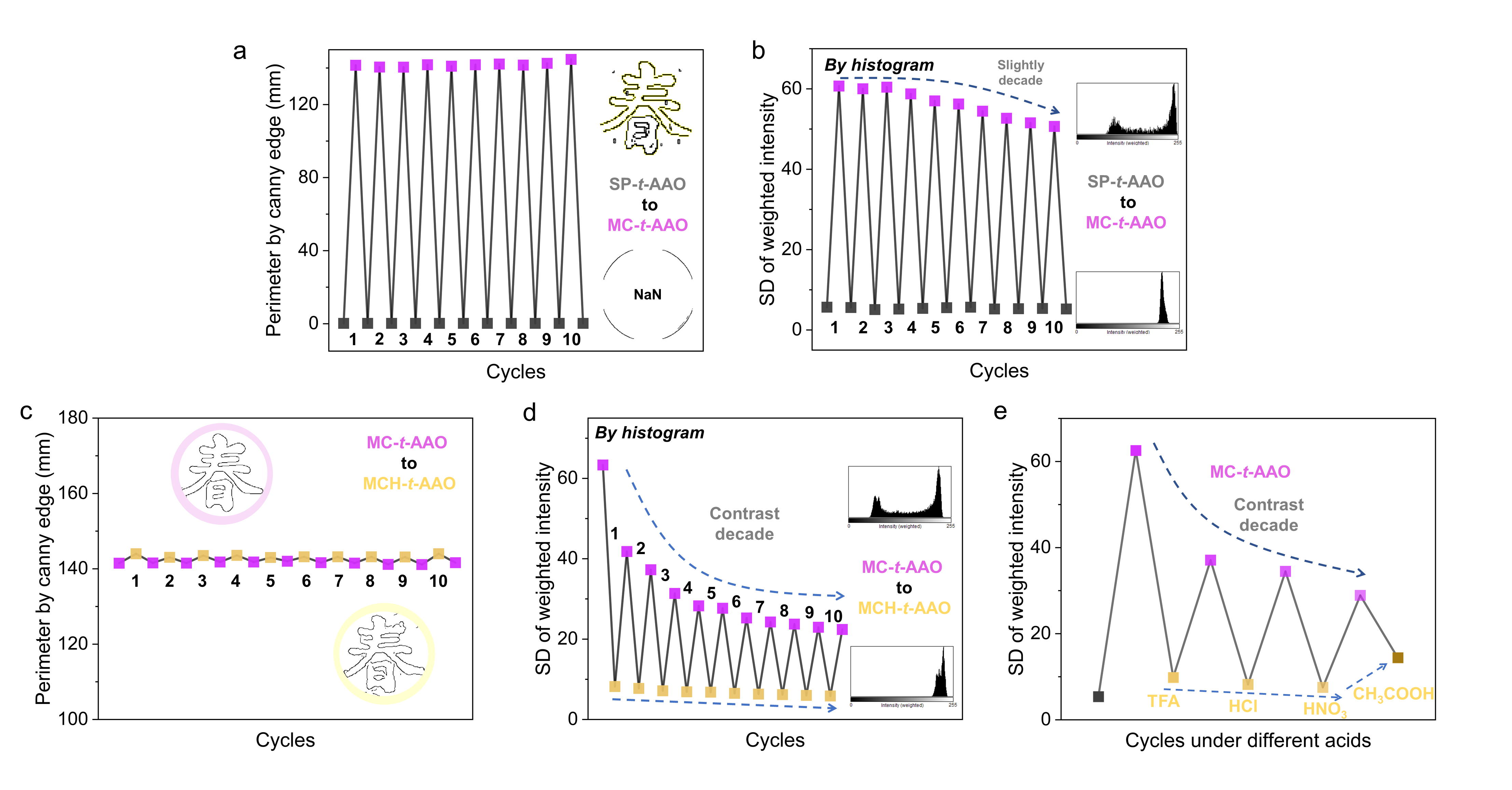

**Figure S10.** Quantitative analyses of the reversibility and reusability of SP-*t*-AAO membranes through image-based Canny edge detection and contrast stability under photochromic and halochromic cycling. (a) Canny edge detection analysis of the perimeter length of the “Spring” pattern on the SP-*t*-AAO and MC-*t*-AAO membranes during repeated UV on/off cycles. (b) Standard deviation of pixel intensity obtained in histogram over repeated UV on/off cycles. (c) Canny edge detection analysis of the perimeter length of the “Spring” pattern on the MC-*t*-AAO and MCH-*t*-AAO membranes during repeated halochromic switching induced by alternating HCl and TEA vapor exposure. (d) Standard deviation of pixel intensity obtained in histogram over acid/base switching cycles (HCl/TEA) (e) Reusability test of a single SP-*t*-AAO membrane subjected to sequential acid vapor treatments (TFA, HCl, HNO_3_, AND CH_3_COOH), with UV regeneration between each acid vapor exposure. The plot shows the standard deviation of the pattern contrast after each switching pair.


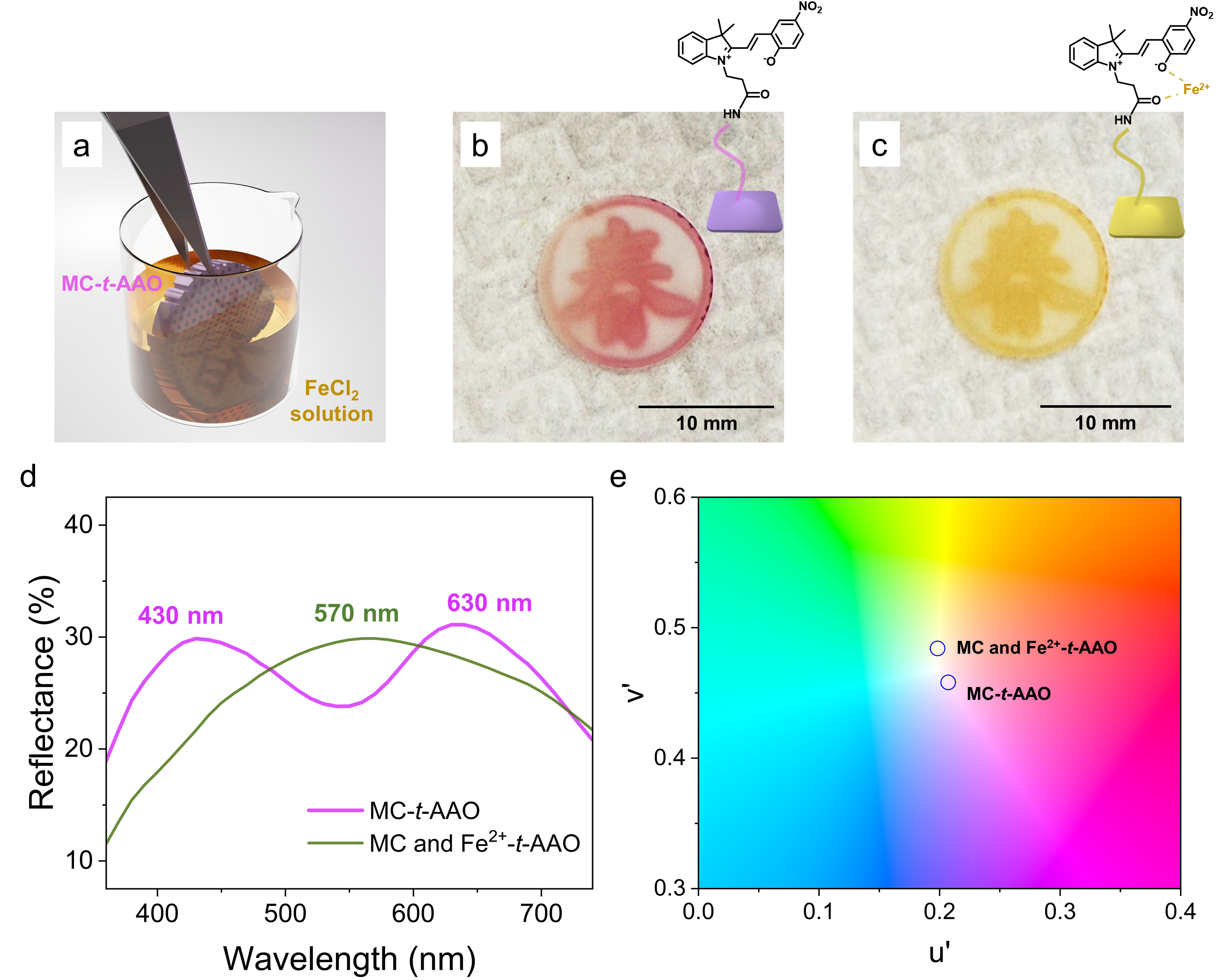


**Figure S11.** Selective Fe^2+^ chelation response of the MC-*t*-AAO membrane. (a) Schematic illustration of immersing MC-*t*-AAO in an FeCl_2_ ethanol solution. (b) Optical image of the MC-*t*-AAO membrane after UV-induced patterning (“Spring”). (c) Image of the same membrane after Fe^2+^ ion exposure, showing a visible color shift toward pale yellow, indicative of merocyanine-Fe^2+^ coordination. (d) Reflectance spectra showing spectral changes before and after Fe^2+^ exposure, with attenuation of the MC-associated bands (~430 and 630 nm) and emergence of a new band near 570 nm. (e) CIE 1976 (u’, v’) chromaticity diagram illustrating the trajectory of color evolution from the pristine state to MC-*t*-AAO and finally to the Fe^2+^-treated state.


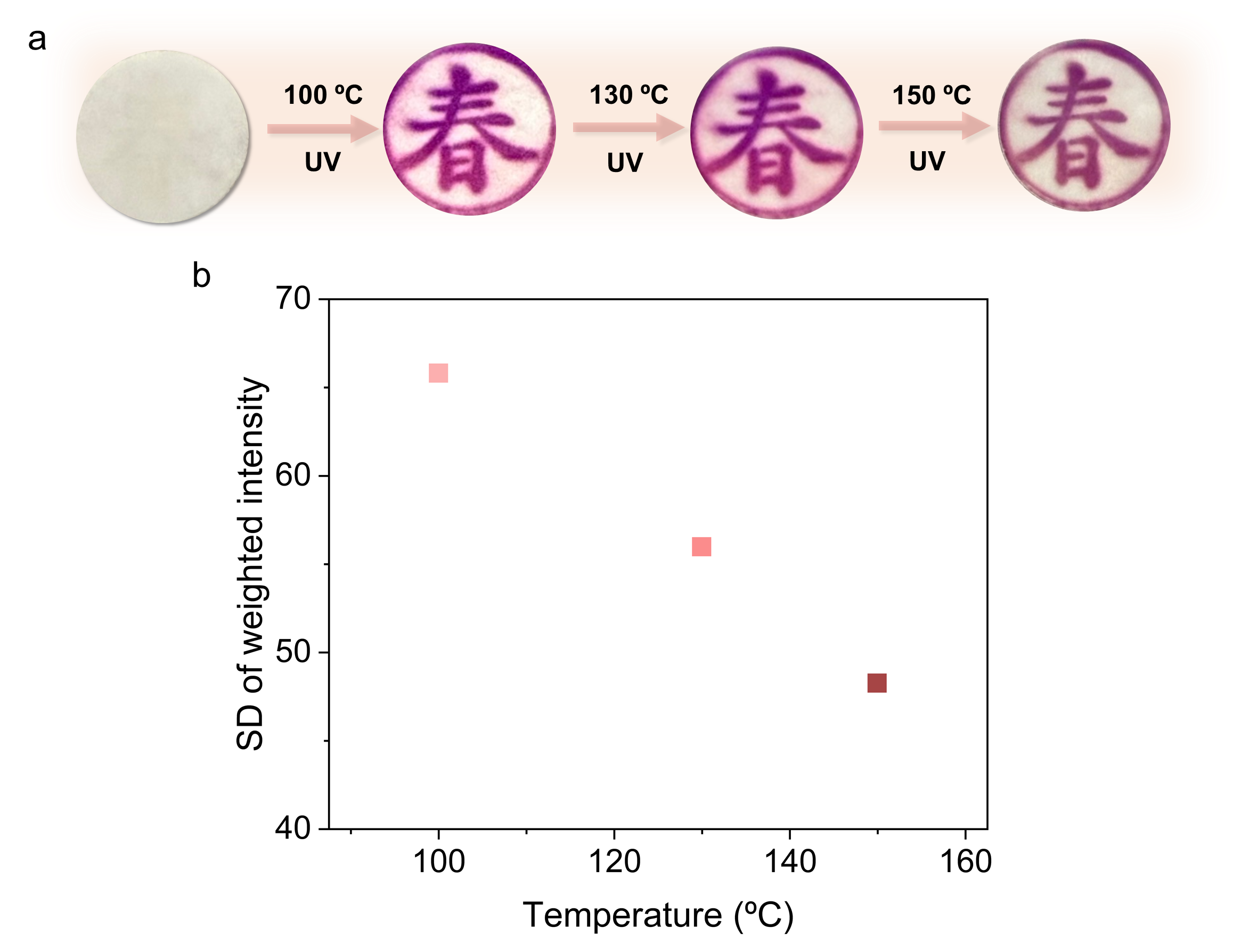


**Figure S12.** Thermal stability of a MC-*t*-AAO membrane under different heating conditions.
(a) Optical images of the MC-*t*-AAO membrane after UV activation and subsequent heating at 100, 130, and 150 °C. (b) Plot of the quantitative analyses of pixel intensity standard deviation across the patterned region as a function of temperature, based on histogram data.


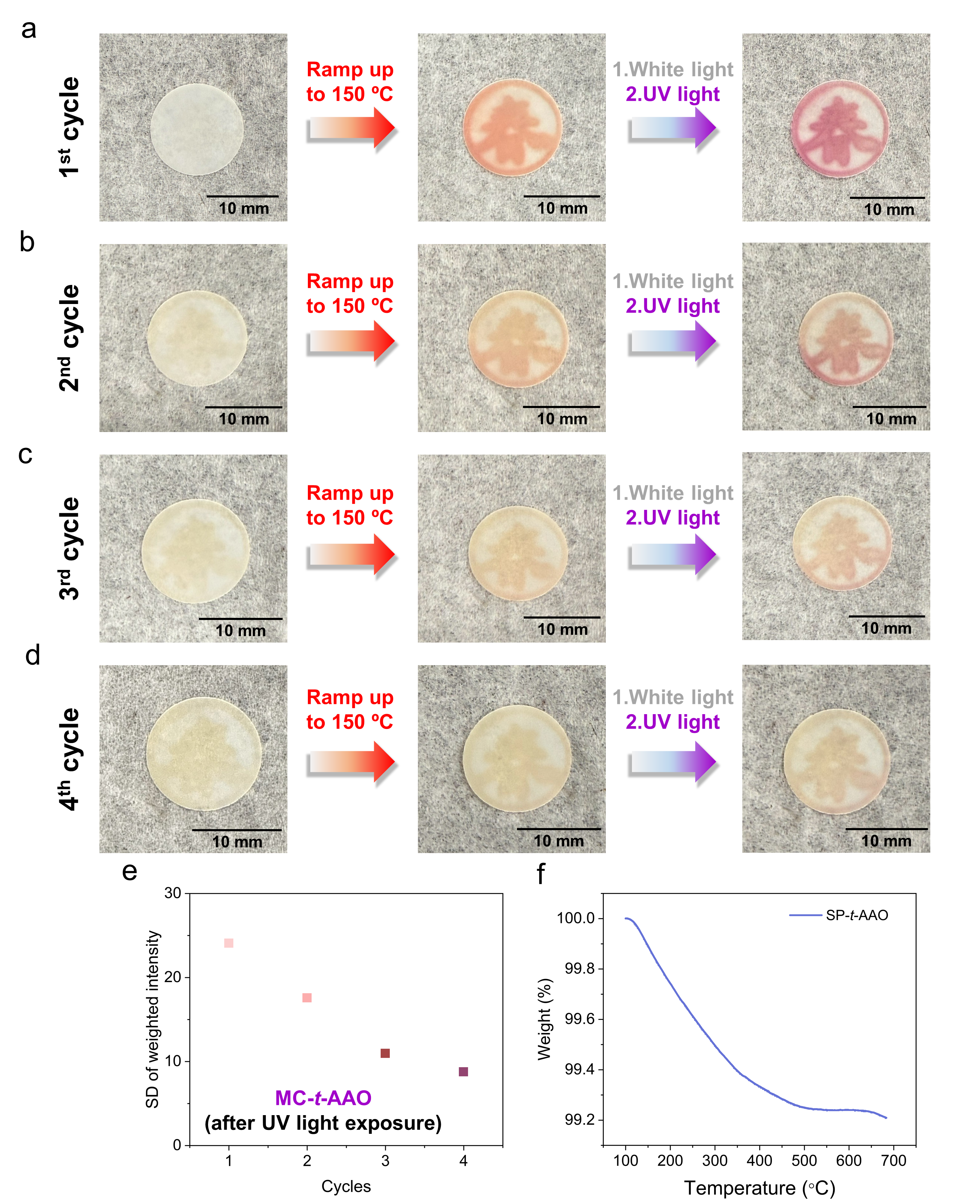


**Figure S13.** (a-d) Thermal cycling tests of SP-*t*-AAO membranes. Each cycle involves ramping the membrane up to 150 °C and holding for 5 minutes, followed by exposure to white light and UV light to observe photo-responsive transformation. After each cycle, the appearances under ambient light (left), thermal-induced coloration (middle), and UV-activated contrast pattern (right) are shown. (e) Standard deviation of pixel intensity (from image histograms) in the UV-activated pattern regions after each thermal cycle. (f) TGA curve of SP-*t*-AAO membrane under air atmosphere, showing onset of weight loss above ~100 °C, indicating partial thermal degradation of organic components.

**Table S1.** Atomic composition (%) of the SP-*t*-AAO membrane before and after thermal cycling (150 °C × 4 cycles)

| **Element** | **C** | **N** | **O** | **S** |
| --- | --- | --- | --- | --- |
| Before  thermal cycling | 22.8 | 2.6 | 72.7 | 1.9 |
| After  thermal cycling | 19.9 | 2.2 | 77.3 | 0.7 |


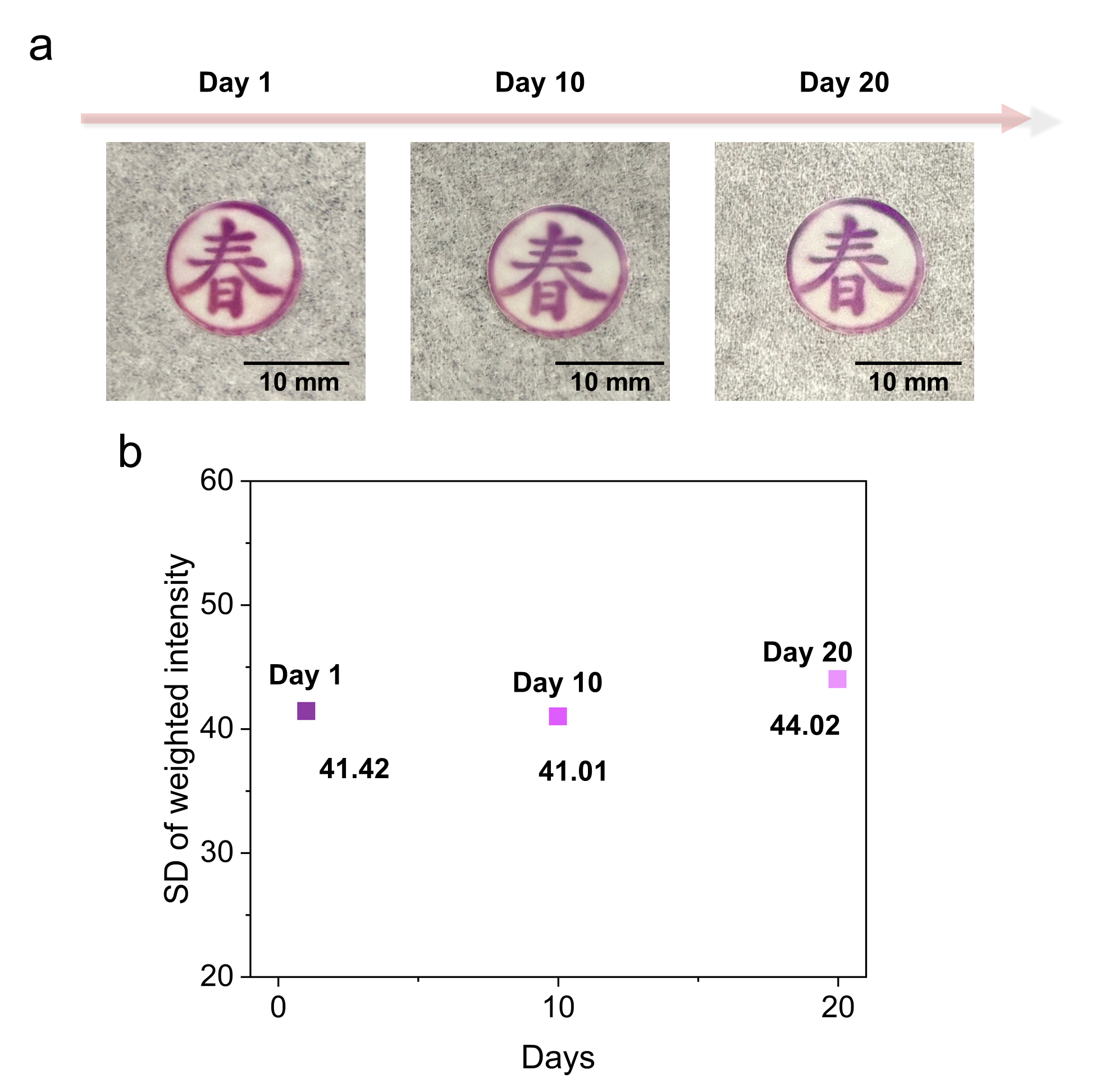


**Figure S14.** (a) Photographs of SP-*t*-AAO membrane with patterns after 1, 10, and 20 days of ambient storage. Prior to each measurement, the sample is irradiated with UV light to trigger the photochromic transition of spiropyran to its colored merocyanine form. (b) Standard deviation (SD) values of the magenta patterned regions over time. The consistent SD values (41.42 on Day 1, 41.01 on Day 10, and 44.02 on Day 20) confirm that the optical contrast is well maintained.


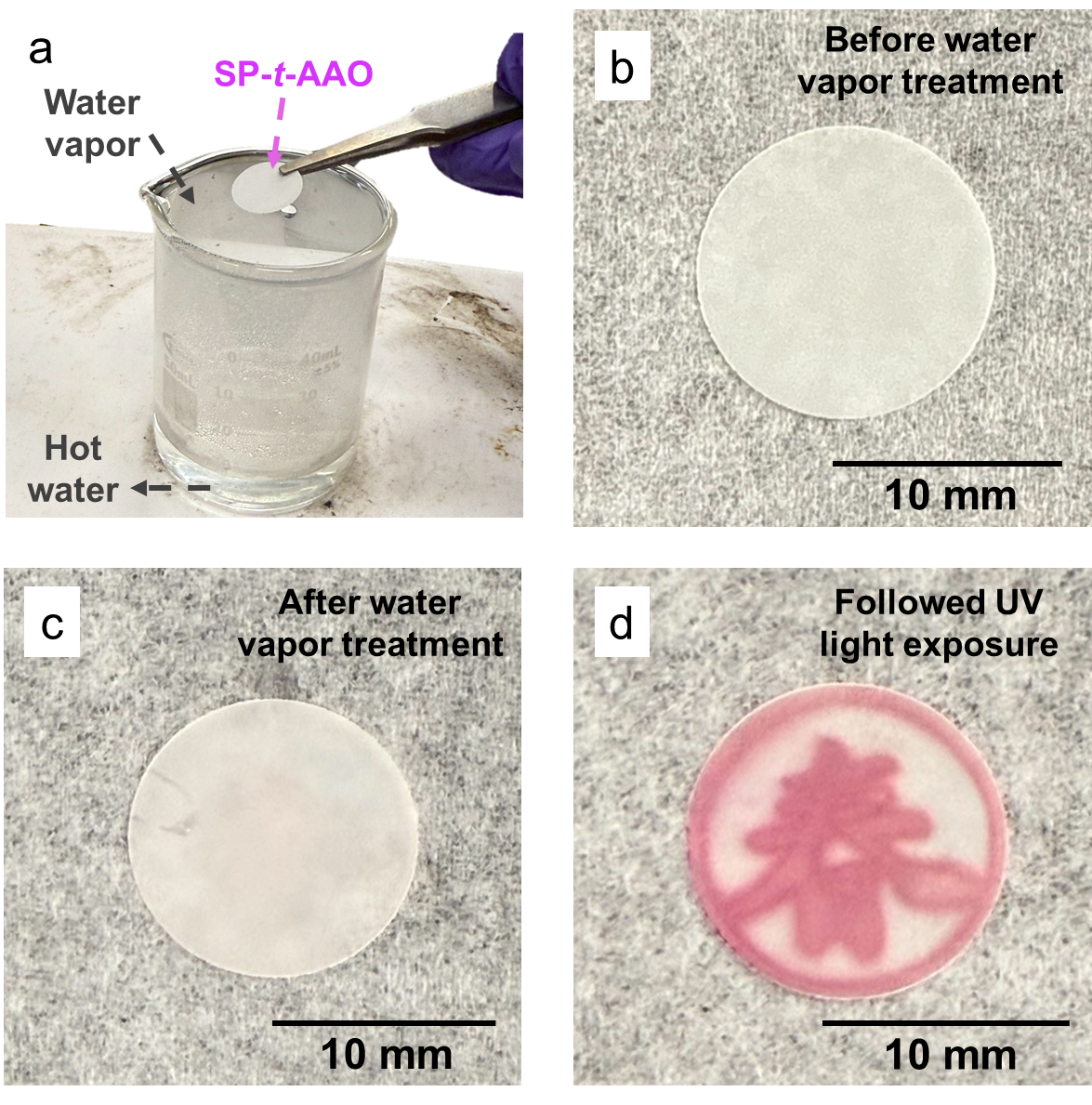


**Figure S15.** Evaluation of an SP‑*t*‑AAO membrane under humid conditions using optical images. (a) SP-*t*-AAO membrane is suspended above hot water to allow exposure to saturated water vapor. (b) SP‑*t*‑AAO membrane before water vapor treatment. (c) SP‑*t*‑AAO membrane after water vapor exposure. (d) Water vapor-treated SP‑*t*‑AAO membrane after subsequent UV light exposure.


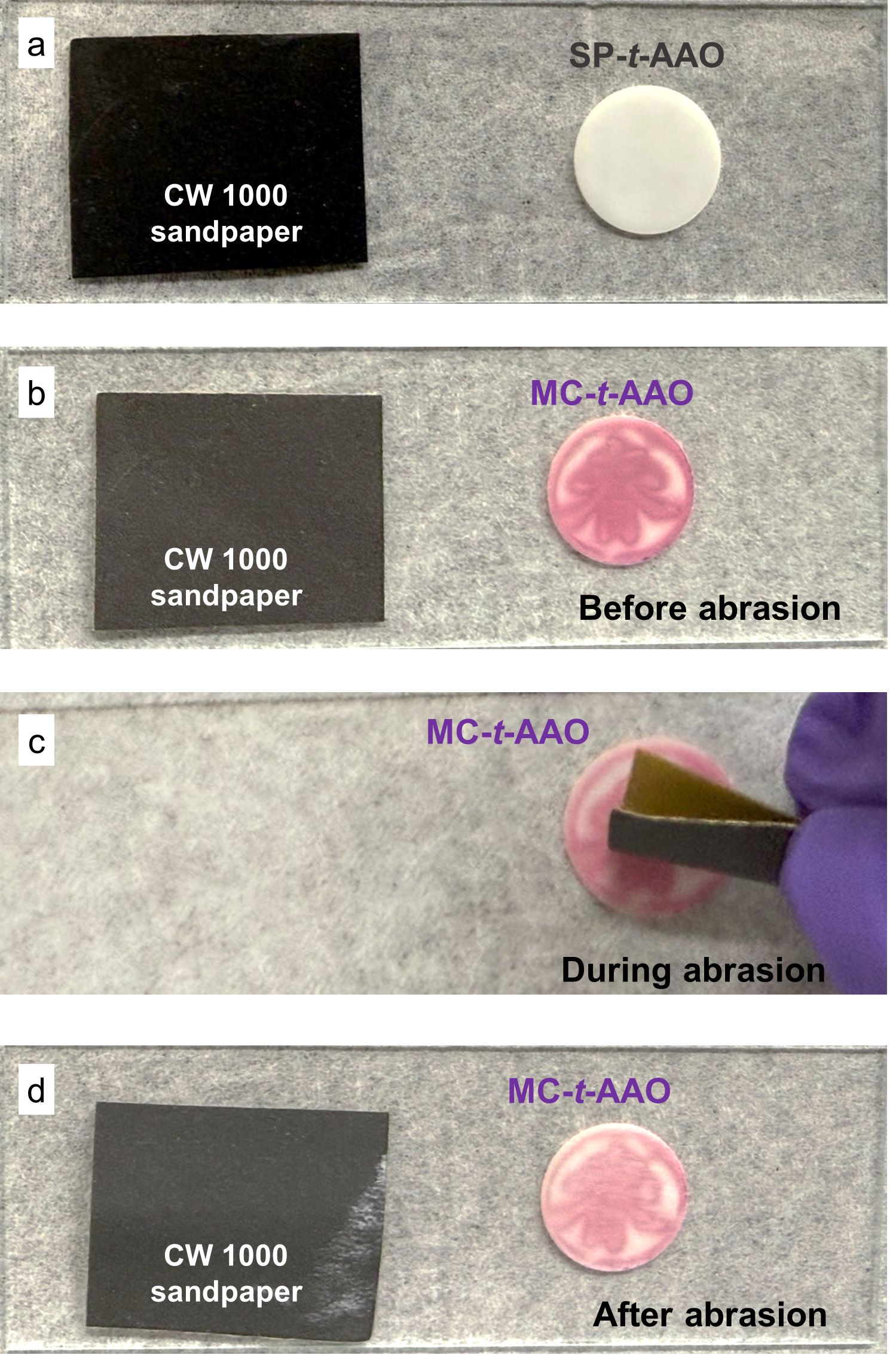


**Figure S16.** Optical images of mechanical abrasion tests on an SP‑*t*‑AAO membrane using CW 1000 sandpaper. (a) Pristine SP‑*t*‑AAO membrane and a sandpaper prior to testing. (b) UV-activated MC‑*t*‑AAO membrane exhibiting a visible pattern and a sandpaper. (c) MC‑*t*‑AAO membrane undergoing surface abrasion using a sandpaper. (d) MC‑*t*‑AAO membrane after surface abrasion.

**Table S2.** Comparison of functional anti-counterfeiting materials and their key performance metrics, including this work and representative studies reported in recent years

| **Reference** | **Material** | **Responsive**  **type** | **Response time** | **Color Contrast (ΔE)** | **Cyclic Durability** |
| --- | --- | --- | --- | --- | --- |
| This work | AAO/Spiropyran (SP-*t*-AAO membrane) | Light/  Acid and Base | Light: 60 s  Acid: 0.4~4 s | Photochromism: 28.99  Halochromism: 27.02  (from CIE coordinate shift) | Photochromism: >10 cycles  Halochromism:  moderate decay in reflectance >10 cycles |
| ^1^ | LDA (lanthanide-doped aluminate) microparticles in polyacrylic acid matrix | Visible/  UV irradiation | 5 min | 0.16~22.83  from CIE coordinate shift | At least 16 cycles |
| ^2^ | Dianthryl sulfoxides doped in PMMA thin films | UV irradiation | 8 s | 24.2 and 2.9 | 2~4 reproducibility for different trails |
| ^3^ | TiO_2_/CsPbBr_3_ fluorescent electrophoretic particles embedded in EPD | UV irradiation | 350 ms | Not reported numerically, butΔE ≈ 17.5-18 from CIE coordinate shift | Stable under continuous operation  (>1000 s) |

**Reference**

(1) Abou-Melha, K. Preparation of photoluminescent nanocomposite ink toward dual-mode secure anti-counterfeiting stamps. *Arab. J. Chem.* **2022**, *15,* 11.

(2) Yuan, J.; Christensen, P. R.; Wolf, M. O. Dynamic anti-counterfeiting security features using multicolor dianthryl sulfoxides. *Chem. Sci.* **2019**, *10,* 10113-10121.

(3) Liu, G. Y.; Wu, X. Z.; Xiong, F.; Yang, J. L.; Liu, Y. H.; Liu, J.; Li, Z. H.; Qin, Z.; Deng, S. Z.; Yang, B. R. Fluorescent, multifunctional anti-counterfeiting, fast response electrophoretic display based on TiO2/CsPbBr3 composite particles. *Light-Sci. Appl.* **2024**, *13,* 11.
